# Explainable multimodal feature fusion networks for Parkinson's disease prediction

**DOI:** 10.3389/fdgth.2026.1771281

**Published:** 2026-02-27

**Authors:** Abishek Ravichandran, Tamilarasi Kathirvel Murugan, Logeswari Govindaraj, Vishal M

**Affiliations:** School of Computer Science and Engineering, Vellore Institute of Technology, Chennai, India

**Keywords:** deep learning, explainable AI, fusion models, gait analysis, handwriting recognition, medical diagnosis, multimodal learning, neural networks

## Abstract

Parkinson's disease (PD) is a progressive neurodegenerative disorder characterized by motor and non-motor impairments, where early diagnosis remains challenging due to reliance on subjective clinical assessments. Recent artificial intelligence (AI)-based approaches have demonstrated promise in identifying subtle PD biomarkers from individual modalities such as speech, gait, and handwriting; however, unimodal systems often fail to capture the heterogeneity of the disease and provide limited interpretability. To address these limitations, this study proposes a multimodal deep learning framework that integrates handwriting, gait, and speech modalities using an early feature fusion strategy for robust and interpretable PD detection. Each modality is processed through a dedicated feature extraction pipeline using deep neural networks, followed by static feature concatenation and classification using an XGBoost model. Model transparency is enhanced using explainable AI (XAI) techniques, including SHapley Additive exPlanations (SHAP) and Gradient-weighted Class Activation Mapping (Grad-CAM), enabling clinical interpretability of modality- and feature-level contributions. Experimental evaluation on benchmark datasets demonstrates that the proposed trimodal fusion model achieves an accuracy of 92%, outperforming unimodal handwriting (91%), gait (90%), and speech (74%) models. The fusion framework attains a macro F1-score of 0.89, an area under the ROC curve (AUC) of 0.95, and an average precision (AP) of 0.96, indicating strong discriminative capability and robustness. Confusion matrix analysis reveals balanced sensitivity (90%) and specificity (89%) across classes. Explainability analysis confirms that handwriting tremor patterns, gait force asymmetries, and speech spectral instabilities are key contributors to PD prediction. These results highlight the effectiveness of explainable multimodal AI in delivering accurate, reliable, and clinically interpretable solutions for early PD detection.

## 1 Introduction

Parkinson’s disease (PD) is a highly widespread neurodegenerative disorder that affects millions of people globally and is characterized by motor impairments which encompass tremors, rigidity, gait problems, handwriting abnormalities, and non-motor symptoms comprising speech dysfunction, cognitive dysfunction, and sleep disorders. Such a complex manifestation predisposes PD as a significant clinical problem, as well as a serious social and economic cost. The conventional diagnosis still depends largely on clinical scale and neurological examination which is mostly subjective and liable to inter-observer reliability, which does not adequately capture the early disease manifestation (1). To address these constraints, scientists have more extensively resorted to artificial intelligence (AI)-based systems that can analyze digital biomarkers in speech, gait, and handwriting, and provide objective and data-driven information about PD detection (2).

Lately, the deep learning breakthroughs have provided amazing results in unimodal diagnostic pipelines. Detection by speech has used spectral and acoustic properties and can be 99% accurate in controlled data sets (3). Inertial measurement unit (IMU) sensor-based (wearable) and motion dynamics-based gait methods have been reported to have good predictive capability distinguishing PD patients and healthy controls with accuracy levels of up to 97% (4). Moreover, handwriting analysis, usually centered on spiral plotting or signature patterns, has been effective with convolutional neural networks with an almost 98% accuracy (5). All these results point to the prospective of digital biomarkers to transform the diagnostics and monitoring processes of PD (6).

In spite of these developments, unimodal methods are severely flawed. Speech-based systems are very susceptible to differences in language, accent, background noise, and recording conditions restricting their generalizability (7). The quality of sensors and compliance of the subject are crucial in gait-based detection and make it challenging to apply in the real world (8). Handwriting-based techniques tend to be based on controlled experiments and not sufficiently large data sets to be able to generalize stably (9). These deficiencies pose issues of clinical scalability of unimodal pipelines (10).

The other significant issue is the interpretability of AI models. A large number of deep learning systems are trained as black boxes, which make predictions without an explicit explanation of how and why a decision will be made. In medical use, where the interpretability of results is paramount, this is an issue (11). The less convinced clinicians of the AI-driven diagnostic systems can be when it comes to the validation of the reasoning behind the results. Recent studies have started incorporating explainability mechanisms such as SHapley Additive exPlanations (SHAP) and Gradient-weighted Class Activation Mapping (Grad-CAM) into PD models that allow visualization of influential temporal or spectral properties but are still underutilized (12). Even very good models without strong interpretability may not find significant clinical use (13).

In an attempt to overcome the failures of the unimodal systems, multimodal fusion has become an interesting solution. Multimodal systems can represent the multifactorial nature of PD through the integration of complementary signals provided by several modalities, i.e., speech, gait, and handwriting (14). This combination enables one modality to counterbalance the noisy, missing, or unreliable modality. Indicatively, gait irregularities when combined with handwriting aspects have produced greater outcomes than either of the modalities (15). Equally, fusion of speech and facial expression has proved to be more diagnostic than speech-only systems (16). In spite of these achievements, a majority of multimodal frameworks are not adaptable and explainable and thus hard to implement in clinical practice (17).

This study is motivated by the fact that the gap between experimental prototypes and clinically viable systems needs to be filled. Parkinson’s disease is heterogeneous in people, symptoms do not all develop and manifest in the same way, and it is therefore important to develop systems which are dynamically responsive to different conditions (18). To measure this variability, a multimodal diagnostic model is needed that is capable of incorporating different biomarkers and is resistant to noise and missing values (19). Moreover, it is essential to incorporate explainable AI to provide transparency and trust among clinicians so that professionals could understand the mechanisms behind predictions which could be tremor-induced handwriting distortions, vocal anomalies, or inconsistencies in a stride (16).

The contribution of this paper is the suggestion of a deep learning-based multimodal system, combining speech, gait, and handwriting as features of Parkinson detection (2). In contrast to conventional models, the framework uses an early feature fusion system to give moving weights on modalities according to reliability to guarantee resilience in noisy or incomplete information conditions (3). It also includes explainable artificial intelligence that includes SHAP and Grad-CAM, which can be easily understood and provide both unimodal and multimodal transparency, which can be more easily interpreted by clinicians (12).

The presented system is strictly tested on benchmark datasets with a wide range of modalities and is thus robust and generalizes to various situations (5). The experimental findings demonstrate that the framework not only performs well in terms of attaining high diagnostic accuracy but also has stability in imperfect conditions and is superior to unimodal and non-adaptive baselines (8). The given approach will build a platform of reliable, interpretable, and scalable AI-assisted healthcare solutions by moving PD detection out of the research stage and into clinical practice (17, 20).

The main contribution of the paper is the creation and implementation of a multimodal explainable deep learning architecture that integrates three modalities, namely, speech, gait, and handwriting, to ensure high and explainable detection of Parkinson’s disease (PD). Moreover, explainable AI (XAI) methods, such as SHAP, Grad-CAM, and Integrated Gradients (IG), are applied in the structure and provide a visual and quantitative explanation of the model decision, which makes the predictions transparent and comprehensible to the clinician. Together, such contributions will address the shortcomings of existing unimodal and black-box models to an AI-based solution that is reliable, scalable, interpretable, and advanced in the diagnosis of PD. This article is presented in a systematic manner, thereby putting the readers through the whole research process. The Introduction section identifies the background, motivation, and problems of early PD detection. The Literature survey section takes into account the current unimodal and multimodal methods and reveals their inefficiency, proving the need of more detailed, interpretable method. The Methodology section elaborates on the proposed model architecture, feature extraction schemes in each of the modalities, and the multimodal fusion process. The Results and discussion section includes the results of the experiment and measures of performance and explainability of unimodal and multimodal. Finally, it ends with a Conclusion and future improvements section where the key findings are summarized, clinical implications are outlined, and extensions such as longitudinal modeling, real-world data integration, and federated learning are provided as improvements in the future.

### 1.1 Problem statement

The heterogeneity of the symptoms and the use of subjective clinical ratings make it challenging to diagnose PD with any degree of dependability at an early stage of the condition. Despite the high accuracy of unimodal AI methods based on speech, gait, or handwriting, they do not tend to be generalized across all populations and have no interpretability, which makes them less appealing to clinical users. In addition, the majority of current models are susceptible to noisy or incomplete data and do not offer much information regarding their decision-making process. This poses a critical gap towards the creation of a robust, adaptive, and explainable multimodal framework that incorporates various PD biomarkers, can withstand imperfect data, and can show interpretable information that can be used to make reliable clinical decisions.

## 2 Literature survey

The given paper is devoted to studying the speech signals to detect PD in the early stages because abnormal alterations are frequently overlooked using traditional measures. The traditional methods were essentially run by hand-cut acoustic features or crude models, which could not find out all the abundance of the vocal signals and were limited in diagnostics. The three approaches that the authors have compared are the transfer learning where pretrained models have been utilized, deep feature extraction through spectrogram, and the classical model of the acoustic model in the instance of the pc-GITA Spanish speech data. Their findings revealed that the discriminatory power of deep spectrogram features was more favorable, better than a maximum of 99.7% accuracy to recognize the classification of certain vowel sounds, and the method was significantly better than the transfer learning methods and other traditional methods. The findings confirmed the spectrogram-based characteristics as a highly effective and reliable tool that can be utilized in the diagnosis of PD in speech (2).

This contribution helped in solving the issue of the existence of irrelevant and redundant features that tend to limit the reliability of voice-based detection of PD. The authors have proposed a combination approach to the evolutionary feature selection and deep learning methods, which can be implemented in a hybrid to increase robustness. On the University of California, Irvine (UCI) voice dataset, they first employed adaptive gray wolf optimization (AGWO) in the process of selecting the most informative predictors, followed by inputs of the sparse autoencoder (AE) being the outputs of these predictors, to do the deep feature representation. Finally, six classifiers were applied to the latent features, and linear discriminant analysis (LDA) gave the best results. This composite pipeline was nearly accurate (95%) in contrast to customary classification techniques. In the paper, the advantages of fusion between metaheuristic optimization and sparse autoencoder learning with regard to the noise in medical data have been successfully demonstrated (1).

The authors of the current study explained the negative aspect of utilizing single or narrow sets of features in the classification of PD using speech data that typically lacks significant Nonlinear Complexity Statistics (NCS). They solved this by devising deep learning models that can reduce a set of numerous features into one structure. Two convolutional neural network (CNN) architectures were put forward each having a parallel-branch design where sets of features were learned separately and then combined. This plan was experimented on the UCI Parkinson data using a leave-one-person-out (LOPO) cross-validation to obtain a realistic generalization over individuals. It was noted that the parallel-branch CNN was better both in terms of accuracy (0.869) and F-measure (0.917) than those trained on single features. The findings revealed that, as anticipated, the combination of multiple vocal parameters brings about considerable outcomes in regard to robustness and performance on biased biomedical databases (18).

The paper has argued that detecting PD at an early stage through motion-based analysis of wearable IMU sensors is probably going to be a challenging project since the symptoms of early PD may only have slight similarities with those of a normal aging person. The objective of the research was to develop models of the neural network, which can discriminate between patients with PD at an early stage and healthy participants by using information about the gait dynamics. A sensor was used to collect data on motion signals and was trained using deep neural networks and assessed based on the presence and severity of the disease. The proposed models demonstrated 99.67% levels of accuracy in detecting early-stage PD, a considerably high level when compared to the conventional ones. The results have indicated the potential of wearable technology to track in the real-life situation, in a non-invasive way, and in a manner that could lead to a suspected diagnosis that may contribute to adding objective and very accurate information to the diagnosis procedure as well as the routine examination of the care consultant The objective of the research was to develop the models of the neural network, which can discriminate between the patients with PD at an early stage and the healthy participants by using the information about the gait dynamics. A sensor was used to collect data on motion signals and was trained using deep neural networks and assessed based on the presence and severity of the disease. The proposed models demonstrated 99.67% levels of accuracy in detecting early-stage PD, a considerably high level when compared to the conventional ones. The results indicate the potential of wearable technology to track in real-world scenarios in a non-invasive approach, which, in turn, allows suspected diagnosis to provide objective and highly accurate depth to the process of diagnosis and the usual evaluation of care providers (4, 21).

The fact that the results of the daily monitoring of the PD progression in the form of wearable sensors are interpretable is often an issue, which causes the application of the most deep learning-based models, known as black box. This issue was taken into consideration in the work, and one of the CNN architectures was offered to get acquainted with the continuous wavelet transformation of the signals registered by a group of IMUs located on various body parts. To be more transparent, the authors took Grad-CAM visualization to highlight the frequency channels and attendance locations that were predominant in the predictions so that the rationale behind the system can be comprehended by clinicians. They discovered that one waist-mounted IMU could achieve a performance of about 0.993 to an almost equivalent performance of a multi-sensor implementation. This finding not only reduced the expenditure and intricacy of deployment but also improved clinical reliability, and thus the system was simple to decipher and use (12).

Finger tapping is also used in PD to determine the disease and also to demonstrate improvement; the conditions of assessment in the case are subjective and imprecise clinical ratings. The authors aimed to overcome it by introducing a computer vision pipeline, where hand-pose estimation processes were employed to obtain the fine motor features through the kinematics of patients and healthy subjects in a video. They have offered a stratified classification method to provide better accuracy to define the presence and phases of advancement of the disease. The suggested system was more accurate than the methods available and provided a more precise and detailed examination of the severity of PD. More than that, the paper also discovered remnants of both the existence of linear and non-linear links between specific movement patterns and the disease development; thus, the clinicians are not restricted by the rating scales anymore, but the creation of more accurate evaluations is possible instead (3).

The purpose of the paper was to address two current issues in the detection of speech-based PD, i.e., lack of interpretability and an unbalanced dataset. The authors have proposed a novel pipeline: the XRFILR, comprising the recursive feature elimination to select the relevant predictors, synthetic oversampling and dataset balancing by using K-Means SMOTE, and explainable AI to give a report of model transparency. This system was introduced to the different classifiers to be tested on different speech databases. The results showed XRFILR to be very precise (96.46%); nonetheless, they established the most significant features of speech in determining the classification. The high reliability combined with the interpretability provided the framework not only with significant predictive power but also with valuable insight into the clinical decision-making and therefore increased its consistency with the real-world PD screening (11).

The paper has suggested a multimodal assessment paradigm, which has integrated the handwriting specimens jointed and organized clinical data because handwriting disorders were found in the majority of patients with PD, yet not reflected in predictive models. The authors came up with Parkinson's multimodal deep network (PMMD): a deep neural network that learns associations between visual handwriting and clinical variables and pays attention where it is required. The framework was found to achieve comparable performance in the identification of the complementary nature of the two modalities by the cross-modal attention. The system was always accurate at 96% that was more than the unimodal approaches that made use of the handwriting or clinical data. The analysis demonstrated the power of multimodal fusion to enhance the diagnosis of PD and the importance of attention mechanism in drawing meaningful associations between the data of various types (6).

Speech and facial movements can provide more detailed information to identify Parkinson's facial dyskinesia (PFD) and have been studied separately or out of phase in most historical studies, thus producing a jumbled picture. The given paper proposed a synchronous fusion model according to which speech is predicted by synchronized movements of lips based on selective attention-based processes. Temporal inferences in vocal features and facial expression were recorded, which enabled the model to realize the synergy of multimodal data better. The approach was contrasted with unimodal and asynchronous fusion baselines and achieved an unweighted average recall (UAR) = 95% which is a substantial improvement over alternatives. The outcome confirmed the fact that time synchronized multimodal system can identify PD with more depth and accuracy (15).

The research problem was the gait-based measurement of PD progression considering the fact that gait is among the most revealing biomarkers in the disease staging. The authors reused PhysioNet data and offered a multimodal system with Perceiver which merged unprocessed ground reaction force data with engineered gait characteristics. Unlike many of its predecessors, the method did not require much hyperparameter optimization and was good at performance. The accuracy of the diagnostic system was 97.3%, and a high correlation coefficient [0.93 with Unified Parkinson’s Disease Rating Scale (UPDRS)] indicated that the system could correlate predictions with clinical ratings. The combination of unprocessed and processed streams of data enhanced the effectiveness of both the diagnosis and progress of the patient with an efficient and scalable process that demonstrated results in PD patients’ disease progression monitoring and diagnosis with machine learning techniques (8, 22).

The current paper explored the critical period of the PD, as the time when the following mild symptoms as compromised sleep cycles or smell disorders are ignored by the clinical test. This issue is difficult because early biomarkers are spread and heterogeneous in the fields and prediction is difficult using the traditional single-modality systems. The authors have addressed this by bringing together a myriad of biomarkers including sleep data, cerebrospinal fluid measurements, imaging, and olfactory features into a cohort of over 500 individuals. It was then compared to 12 conventional algorithms, and a deep learning model was found to work significantly better with an accuracy at 96.45%. It was interesting to note that the deep model offered the most predictive performance besides the most discriminative biomarkers that could possibly be employed to assist in the explanation of early disease processes. The study proved the significance of multimodal fusion and the deep learning representation in enhancing sensitivity during early PD detection phases (5, 23).

The complexity and multidimensionality of the signs of PD were also discussed in this paper where no single model could be used to generalize to various manifestations of patients. The authors presented a stacking ensemble model whereby support vector machine (SVM), gradient boosting, and logistic regression are incorporated in the new model in a complementing manner. The ensemble was trained and tested on two benchmark datasets, with the cross-validation procedures being very strict to ensure overfitting is limited, and was also probability-calibrated to yield the correct diagnostic confidence scores. The accuracy of the ensemble was 96.18 which is greater than that being achieved by the base learners. Through the analysis, hybrid datasets were also indicated to be better in addition to straight classification accuracy, more dependable estimations of probabilities, and, consequently, more practical in clinical decision-making in PD detection (7).

To improve the accuracy of the speech-based PD detection, we suggested a hybrid deep neural network, PD-Net, implementing the merits of different spectral features. It was explained by the fact that the cues associated with PD are observed in both fine-grained timbral scales and in more coarse spectral energy scales, but most models consider either one of these two classes of features. PD-Net further used Mel-frequency cepstral coefficients (MFCCs) and Mel spectrograms in a CNN–long short-term memory (LSTM) network and used multi-head attention to estimate the strength of the channel and time step effects. The system is demonstrated to be highly accurate (nearly 99%), and this is greater compared to the single-feature pipelines that demonstrate that the attention-based dual-spectral fusion system can offer discriminatory representations of the PD-related vocal changes (24).

In this paper, the processing of MRI images was reconceptualized to identify PD using graphs in addition to using purely conventional convolutional networks. The challenge is that the neuroimaging data are not very rich and larger networks are prone to overfitting and not good generalization. To get around this, the authors removed the salient points in MRI slices and tabulated these as compact graphs which contained the important structural information. A multi-level graph neural network (GNN) which incorporated pooling and attention layers was also introduced to preserve significant topology and eliminate spurious noise with sparsity-sensitive pooling and attention. This method compared to conventional CNNs and traditional GNNs is said to have had a superior performance and a superior generalization of small training sets. The benefit of the study was that it offered a model architecture which is consistent with the structural priors present in neuroimaging and offered a consistent and data-efficient way of identifying PD using neuroimaging (19).

This systematic review considered 87 articles to evaluate the current state of multimodal deep learning to diagnose PD, integrating all the evidence found in the given areas: speech, gait, facial expression, handwriting, imaging, and physiological recording. This review discovered that the multimodal systems are superior to the unimodal systems as they possess distinct benefits as they are complementary phenotypes as far as none of the modalities can have. It has also identified some of the existing barriers that are being listed to make progress, including small and isolated datasets, non-uniform data recording processes, no elucidation of deep models, and no external validation with clinical setups. The authors have proposed that further work should be done to develop bigger shared benchmarks, more powerful reporting frameworks, and in applying explainable AI framework to reduce the gap between research prototypes and clinically qualified diagnostic systems (13).

Another well-known PD characteristic is the impairment of handwriting, although most of the handwriting datasets lack any significant sample sizes, and consequently the effectiveness of single deep learning models cannot be offered. This issue is resolved in this paper by complementing handwriting data with three distinct sources and training with a wide range of architectures with different representational abilities. The authors failed to present one of these networks; they took intermediate-level features of such CNNs and fed them into an SVM classifier to show superior results. The set was able to record a range of handwriting characteristics using stroke forms, micro-variations which occurred in tremor and rhythms. The system achieved an impressive result of 99.35% and also demonstrated the effectiveness and efficiency of feature-level fusion of heterogeneous CNNs in small samples of handwritten-based PD identification (9).

Our proposal was a deep learning-based methodology, the Multi-Variant Stacked Autoencoder (MVSAE) as a novel framework aiming at the incorporation of numerous domains of PD indicators instead of individual-symptom identification. This was based on the arguments that the analysis of voice, motor, and non-motor features independently lacks important correlations among and across domains and, therefore, hinders predictive validity. MVSAE modeled these attributes together and found small shared representations of reduced redundancy with no loss of disease-relevant variation. In a sequence of experiments, the model did better than other simple classifiers by differences up to 5–10 percentage points and showed that cross-domain embeddings actually are more useful than isolated feature representation of recognizing PD. The study merits are that the latent PD signatures could be detected using holistic, integrative representation learning and appear to be more representative of the heterogeneity of this complicated disease (14).

This contribution coined the new definition of stroke segmentation as a physiologically significant segmentation strategy that enables positioning the new PD detection roadmap on the scene. The system could model strokes based on the beta-elliptical technique of breaking down handwriting into perceptually significant units and was followed by the fuzzy perceptual detector which could extract features that were resistant to tremor noise and intra-writer variation. It was then fed with the characteristics of these features into a bidirectional long short-term memory (BLSTM) network to take into account temporal relationships between the individual strokes. The PaHaW handwriting corpus was experimented upon, and the system performed much better than the current handwriting-based PD classifiers. The combination of physiologically plausible stroke segmentation and sequence modeling actually improved the accuracy and provided a feasible form of passive screening using a tablet, either in a clinical or home-based environment (10).

This article was a resolution to the issue of tuning the long short-term memory (LSTM) networks by applying them to the gait time-series information, and these are sensitive to the hyperparameters. The authors suggested a variant of the particle swarm optimization process modified to run a hyperparameter search algorithm automatically and applied it to PhysioNet gait data sets. The optimized LSTM was 89.92% accurate and higher than the unoptimized models and other optimization strategies. To improve the transparency of the study, SHapley Additive exPlanation (SHAP) analysis was employed to understand the most significant temporal gait features in prediction, and it was possible to see the physiological rationale of the model decisions. The study demonstrated that principled optimization with *post hoc* explainability can obtain an effective and interpretable differentiation system of gait-based PD detection (16).

The paper (25) proposed a non-invasive PD prediction method leveraging voice inputs, demonstrating that speech-based biomarkers can effectively predict disease progression. This approach complements multi-omics and clinical data-driven strategies, highlighting the value of integrating diverse data sources for early and accurate PD risk assessment.

In the Parkinson’s Progression Markers Initiative (PPMI), 12 datasets of single and multimodal ML pipelines were compared on PD analysis. They also investigated different kinds of classifiers besides feature selection methods so as to examine the impact of the source of data on them. To a surprise, the results proved that not all simple, deep, or complex models are effective as in scenarios when there is good quality data and features are correctly performed, a simple linear SVM will get the perfect accuracy. However, with higher heterogeneity of the data, multimodal fusion was more resistant and capable of generalizing across a broader range of patients. There were also compressive feature attribution with predictors in the motor, olfactory, cognitive, and sleep domains. This cumulative finding suggested that both simplification and multimodal fusion might be applicable in the detection of PD: The linear models might be sufficient under the conditions of high quality of the data, but multimodal fusion might be helpful under the conditions of higher variability levels.

Current PD detection systems are based mostly on unimodal methods of detecting speech, gait, or handwriting as an independent entity, which restricts their resilience, generalizability, and clinical reliability. The speech-based models are also influenced by language, accent, and noise differences, gait-based models are influenced by sensor quality and user compliance, and handwritten-based models are sensitive to controlled environments and large datasets. In addition, a majority of deep learning models deployed in these studies are black boxes and thus provide limited insight or comprehension of the manner in which they arrive at a decision and therefore are not easily trusted and utilized by clinicians. These unimodal approaches also cannot capture the heterogeneity of the PD symptoms and cannot handle noisy or incomplete data. The proposed multimodal explainable model addresses these weaknesses by incorporating complementary biomarkers of speech, gait, and handwriting of feature fusion which is resistant to data flaws and cross-domain interaction. Besides that, explainable AI (SHAP, Grad-CAM, and Integrated Gradients) is also embedded and offers transparent, physiologically interpretable explanations of predictions made by a model, allowing clinicians to know which features affect a diagnosis. It leads to a single, strong, and interpretable system of PD detection that is more accurate and bridges the gap between the experimental research on AI and its use in practice.

While previous studies have applied unimodal approaches for PD detection using speech, handwriting, or gait separately, they often do not integrate heterogeneous modalities for comprehensive assessment. Similarly, multimodal frameworks reported in focus on feature-level fusion but lack explainability or prospective validation. In contrast, the proposed framework combines three complementary modalities (speech, handwriting, gait) with a unified deep feature extraction and explainable AI pipeline, providing improved interpretability and decision support capabilities. This positions our work as a novel contribution bridging multimodal integration with clinical applicability, beyond the scope of prior studies. Table 1 summarizes the key attributes of relevant studies on PD detection, highlighting the modalities used, classification strategies, and explainability techniques.

**Table 1:** Summary table of key attributes of research papers.

Reference	Dataset	Methodology	Results and discussion	Metrics	Application	Strength	Limitation
(2)	pc-GITA Spanish speech dataset (phonations + read speech)	Compared transfer learning, deep spectrogram features + ML, and classical acoustic features	Deep features outperformed others; accuracy up to 99.7	Accuracy, comparison with the existing literature	Early PD detection of speech	High accuracy, generalization by speech tasks	Language-specific dataset limits generalization
(1)	UCI PD voice dataset	Adaptive gray wolf optimization for feature selection + sparse autoencoder representation learning	Best LDA model achieved a top of 95%	Accuracy, cross-validation performance	Voice-based PD classification	Combines feature selection and deep representation	Single dataset; can overfit to particular voice patterns
(18)	UCI PD voice dataset	CNN models with concatenated or parallel-branch features	Parallel CNN outperformed Accuracy 0.869, F-measure 0.917	Accuracy, F-measure, MCC	Multiset feature speech-based PD detection	Improved handling of imbalanced data	Moderate absolute accuracy compared to the latest
(4)	IMU gait data of PD patients and controls	Neural networks on motion data	Early-stage detection accuracy up to 99.67%	Accuracy	Wearable-based PD detection	Very high early-stage detection accuracy	Small sample size
(12)	IMU data of 100 subjects	CNN wavelet transforms and Grad-CAM pruning features	Single waist sensor achieved 98.01% accuracy, AUC 0.9981	Accuracy, AUC	Best wearable PD monitoring	High accuracy with minimal sensors	Focus on walking only; other PD signs may escape
(3)	Videos from 90 subjects	Hand-pose estimation + tiered classifier	Outperformed prior severity classification methods	Accuracy, feature trend analysis	Video-based severity in PD	Detection of fine-grained severity change	Depends on video quality and a controlled environment
(11)	Speech dataset	XRFILR pipeline: RFE + LR, K-Means SMOTE, explainable ML	Accuracy 96.46%, interpretable feature importances	Accuracy	Explainable speech-based PD prediction	Interpretable results for clinicians	Only to speech features
(6)	Handwriting/drawing images + clinical data	Cross-modal attention fusion deep learning	96% accuracy; outperformed baselines	Accuracy	Multimodal PD detection	Integrates complementary modalities	Requires both data types for best results
(15)	Video of speech, audio, and lip movement	Attention synchronized bimodal fusion	UAR 95% with synchronous fusion	UAR	Multimodal audio-visual PD detection	Improves over unimodal/asynchronous	Require good quality synchronized data
(8)	PhysioNet gait dataset	Perceiver architecture on GRF + gait features	UPDRS MAE 2.23; PD diagnosis 97.3% accuracy	MAE, RMSE, CC, Accuracy, AUC, Sensitivity, Specificity	Remote gait-based PD diagnosis and severity scoring	High accuracy for both diagnosis and severity	Lab-collected gait may differ from daily life
(5)	183 healthy, 401 early PD (premotor features)	Deep learning model vs. 12 ML/ensemble methods	Deep model has the highest average accuracy, 96.45%	Accuracy	Premotor biomarker-based early PD detection	High accuracy across diverse indicators	Relatively small dataset size
(7)	Two benchmark PD datasets	Stacking with SVM + GB for features, LR classifier	Accuracy 94.87% (AUC 90%) and 96.18% (AUC 96.27%)	Accuracy, AUC	Improved PD diagnosis from standard datasets	Strong performance across datasets	Benchmark datasets may not reflect real-world noise
(24)	Italian vocal audio dataset	MFCC + mel spectrogram via CNN + LSTM + attention	99% accuracy	Accuracy	Speech-based PD detection	Captures both spatial and temporal patterns	Single-language dataset
(19)	PPMI MRI slice data	Image-to-graph construction, multi-level GNN, sparsity pooling	Outperformed CNN/GNN baselines	Accuracy	MRI-based PD diagnosis	Efficient graph construction; reduced overfitting	MRI preprocessing still required
(13)	87 reviewed studies	Survey of DL on gait, limb, speech, and facial expression	Multimodal > unimodal; gaps in interpretability	N/A	Comprehensive review of PD DL approaches	Broad coverage across modalities	No experimental results
(9)	Three PD handwriting datasets	Multiple CNNs, early fusion of features, SVM	99.35% accuracy	Accuracy	Handwriting-based PD detection	Superior to single CNNs	Requires extensive data augmentation
(14)	Multi-feature PD dataset	Four SAE variants for multi-attribute learning	10% better than MANN, GAE, UMLBD	Accuracy	Multi-attribute PD prediction	Handles diverse PD features	Limited external validation
(10)	PaHaW and new Arabic handwriting dataset	Beta-elliptical stroke segmentation, fuzzy features, BLSTM	Outperformed existing handwriting-based PD systems	Accuracy	Online handwriting PD detection	Novel stroke + fuzzy feature combo	Language-specific dataset
(16)	PhysioNet gait dataset	LSTM optimized with modified PSO	89.92% accuracy; SHAP for feature importance	Accuracy	Gait-based PD diagnosis	Metaheuristic tuning improved performance	Performance still below some multimodal models
(17)	12 PPMI datasets	Single/multimodal ML, majority voting labeling	SVM 100% acc; ANN 91.41% with selected features	Accuracy	Identify PD and key causal factors	Identifies influential features	May overfit due to very high accuracy

MCC, Matthews correlation coefficient; RFE, recursive feature elimination; MAE, mean absolute error; RMSE, root mean square error; GRF, ground reaction force; GB, gradient boosting; MFCC, Mel-frequency cepstral coefficients; DL, deep learning; SAE, stacked autoencoder; MANN, multimodal artificial neural network; GAE, graph autoencoder; UMLBD, unified multimodal learning-based diagnosis; PSO, particle swarm optimization; ANN, artificial neural network.

## 3 Methodology

The suggested methodology combines three complementary modalities, such as gait, voice, and handwriting, into a single multimodal diagnostic tool for PD. Individual modalities receive their own specialized feature extraction pipeline: Gait signals are processed using temporal convolutions with dilations and autoencoding, voice data are processed using EfficientNet-B0 with log-Mel spectrograms, and spiral handwriting images are processed using ResNet-50. Such pipelines record modality-dependent biomarkers including stride anomalies, phonatory errors, and tremor-related anomalies. Features are normalized, compressed through global average pooling, and converted into small latent vectors to make them comparable across domains. SHAP, Integrated Gradients, and Grad-CAM support the interpretability of their prediction at the modality level and allow clinical professionals to reverse engineer the way their model arrives at its prediction. After unimodal extraction, the feature vectors are then fused together in a combined multimodal representation that includes domain-specific and cross-domain information. This combination embedding is propagated by XGBoost to get the resultant predictions. Optimization of the model is done with the help of cross-entropy loss, and interpretability is maintained through the calculation of SHAP values of the fused vector. Not only does this multimodal fusion outperform unimodal systems in accuracy of classification, but it also provides clinically relevant and interpretable information on heterogeneous symptoms of PD.

### 3.1 Gait feature extraction pipeline

**Figure 1:**
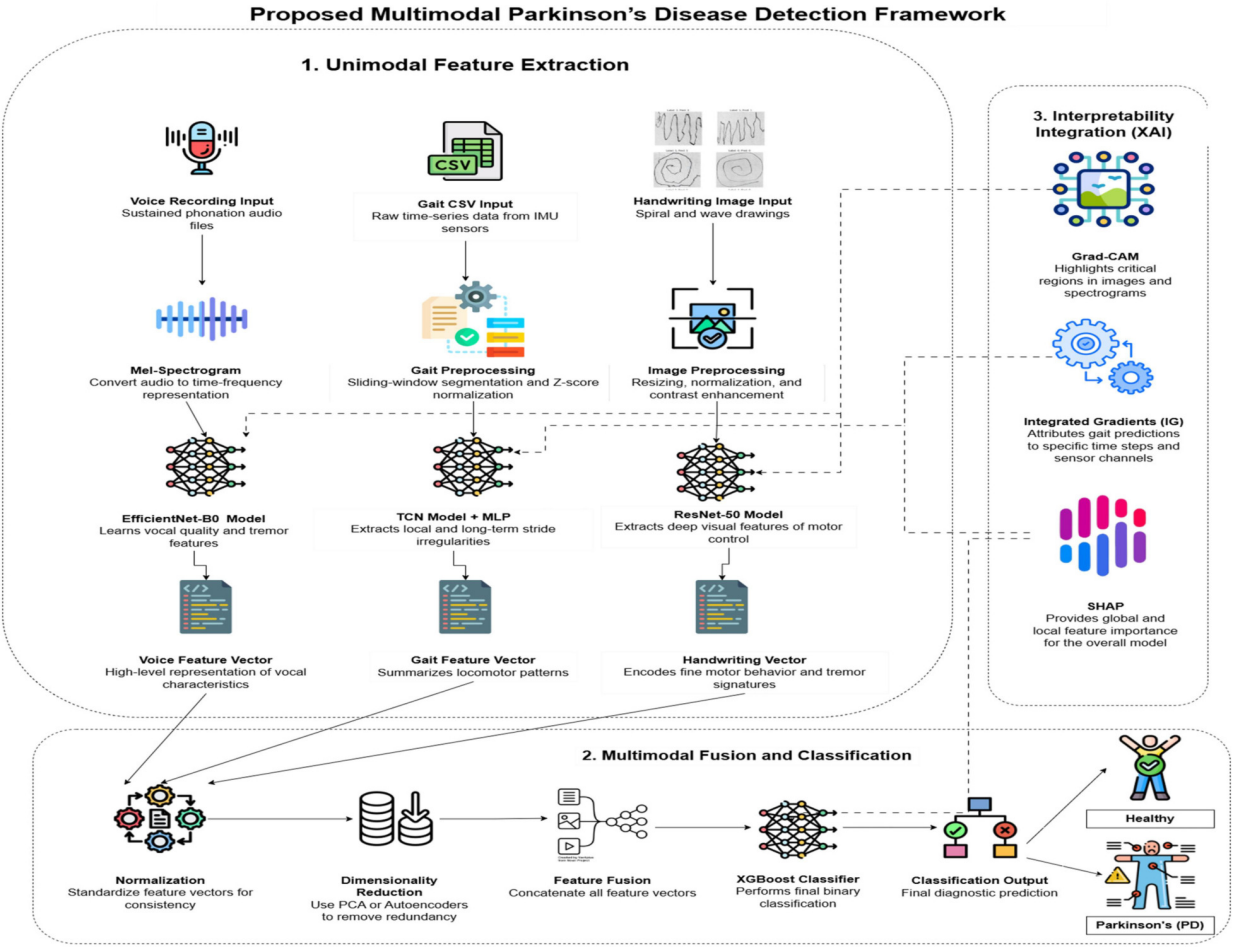
System architecture of the proposed framework.

As discussed in Figure 1, the initial step of the pipeline is to put the raw gait signals that are usually of different lengths and magnitudes across subjects into a uniform form. All recordings are broken up into temporal blocks of certain fixed lengths of 2 s (200 samples at 100 Hz) and still maintain the temporal continuity. In case one of the segments is shorter, it is zero-padded to preserve the dimension. A normalization of these windows is then made based on *z*-score scaling which rescales each feature by removing the mean and dividing by the standard deviation. This modification makes sure that the different sensors or trials with different absolute magnitudes do not overtake the training process. This normalization formula can be expressed as in Equation 1:

$$V_{\mathrm{norm}}=\frac{q-\mathrm{mean}}{\mathrm{SD}}$$ (1)

where $V_{\mathrm{norm}}$ is the normalized value, *q* is the raw input value, mean is the average of the windowed interval, and SD is the standard deviation of the windowed interval.

This is applied to normalize the data so that its variance is 1 and its mean is 0, which improves the stability of training and makes the features of other sensors comparable.

The second step is to use temporal convolutions to get local fine gait dynamics. Convolution is nothing but a weighted average of time samples around it, which has gone through a non-linear activation (ReLU). This enables the network to automatically learn filters which identify stride changes, tremor bursts, or asymmetries. The temporal convolution at a time step is of a basic form which is expressed as in Equation 2:

$$\begin{array}{cccc} & h_{t}=a\left(\overset{k-1}{\underset{i=0}{\sum }}p_{i}.e_{l+i}+(\mathrm{bias})\right) & & \end{array}$$ (2)

where $p_{i}\;$ are kernel weights, $e_{l+i}$ are input samples within the convolutional window, bias is a bias term, and *a* is the activation function.

Instead of using local patterns, dilated convolutions are used, in which gaps are inserted between sampled inputs. This increases the receptive field (but does not raise the cost of computation) to allow the network to trace longer gait cycles. The convolution of the layer is dilated. *l* is formalized as in Equation 3:

$$\begin{array}{cccc} & h_{t}^{\left(l\right)}=f^{\left(l\right)}\;(h_{v-c}^{\left(l-1\right)},\ldots ,h_{t}^{\left(l-1\right)}) & & \end{array}$$ (3)

where *c* is the dilation factor, meaning that the filter skips every *c* samples from the previous layer.

By stacking layers with different dilations, the model can capture both short-term stride irregularities and long-term gait cycles, as defined in Equation 4.

$$\begin{array}{cccc} & y_{t}=h_{t}^{\left(l\right)}+p(i_{d}) & & \end{array}$$ (4)

where $p(i_{d})\;$ is the residual mapping of the input.

This mechanism ensures that the network does not lose fine-scale gait information while learning higher-order abstractions.

The model generates rich temporal features for the maps after several convolutional layers. These maps are, however, different in length depending on the size of the window and convolution parameters. Global average pooling is used to compress this onto a fixed-length vector that is a representation of the gait segment. This is utilized to compute the average activation at each time step giving a compressed embedding. The operation is constituted as in Equation 5:

$$\begin{array}{cccc} & \;f=\frac{1}{Q}\overset{Q}{\underset{t=1}{\sum }}h_{t} & & \end{array}$$ (5)

where *Q* is the temporal length of the segment and *f* is the resulting embedding.

This latent vector captures dominant gait dynamics (e.g., stride frequency or imbalance) in a low-dimensional space, making it suitable for clustering and classification.

The model is trained, as an autoencoder, to make sure that important information is not lost in the learned embedding. The encoder is fed by inputs and transforms them into embeddings, and the decoder tries to rebuild the original input signals based on the embeddings. The reconstruction goal causes the embeddings to capture gait features of significance as opposed to noise. This is formalized as in Equation 6:

$$\begin{array}{cccc} & {\hat{m}}_{k}=g_{d}(u) & & \end{array}$$ (6)

where $g_{d}$ is the decoder function, *u* is the latent embedding, and ${\hat{m}}_{k}\;$ is the reconstructed signal.

Minimizing the difference between ${\hat{m}}_{k\;}$ and the true $m_{k}$ ensures that embeddings encode physiologically relevant gait dynamics.

After generating embeddings, they are clustered into groups to determine natural clusters of gait patterns. The redundancy is eliminated using dimensionality reduction (through PCA) after which the K-means clustering is carried out.

To estimate the best number of clusters, *K*, the silhouette score is calculated which weighs the intra-cluster cohesion and inter-cluster separation. The definition of this metric is shown in Equation 7:

$$\begin{array}{cccc} & s=\frac{c-d}{\max(c,d)} & & \end{array}$$ (7)

where *d* is the mean distance of points of the same cluster and *c* is the mean distance between the current cluster and the other nearest cluster.

When the silhouette score is high, the clustering will be done in a better way, and it helps to find meaningful subgroups such as the case of healthy gait and the case of Parkinsonian gait.

A shallow neural network classifier is trained on the embeddings with pseudo-labels obtained through the process of clustering to transform the unsupervised cluster assignments into a predictive tool. The softmax function is the calculation of the probability of classes by the classifier, as shown in Equation 8:

$$\begin{array}{cccc} & {\hat{m}}_{l}=\frac{\exp(\;p\cdot g_{l}+b_{l})}{\sum_{\;j=1}^{l}\exp(\;p\cdot g_{l}+b_{l})} & & \end{array}$$ (8)

where *g_c_* and *b_c_* are classifier parameters, *l* is the total number of clusters (classes), and ${\hat{m}}_{l}\;$ is the probability that input *p* belongs to class *c*.

The model is optimized by reducing the non-entropic loss which penalizes variance between actual labels and probabilities. This is defined in Equation 9:

$$\begin{array}{cccc} & L=-\overset{C}{\underset{c=1}{\sum }}o_{h}\log({\hat{y}}_{c}) & & \end{array}$$ (9)

where *o_h_* is the actual text (one-hot encoded).

This guarantees that the classifier learns decision boundaries that match gait clusters and thus it can classify unseen gait segments with a lot of strength.

SHapley Additive exPlanations (SHAP) is used to provide interpretability. SHAP is based on the cooperative game theory, and the score of significance to each feature is calculated by measuring its marginal contribution to any feature subset. This is regularized in Equation 10:

$$\begin{array}{cccc} & \;p_{i}=\underset{S\subseteq F\{l\}}{\sum }\frac{|A|!(|W|-|A|-1)!}{|W|!}\;[w(A\cup \{i\})-w(A)] & & \end{array}$$ (10)

where *p_i_* is the importance of feature *l*, *W* is the full feature set, *A* is a subset excluding feature *l*, and w(⋅) is the model prediction.

As a complementary interpretability technique, Integrated Gradients (IG) explains the prediction of a neural network using the input features of the network by summing gradients along a linear path between a baseline (e.g., zero input) and the actual input. IG contrasts with SHAP, which is a combinatorial-based method, and operates on a direct measure of the sensitivity of outputs to the change of input values. Equation 11 defines the attribution as follows:

$$\begin{array}{cccc} & IG_{i}(k)=(k_{i}-k_{i}^{\prime })\cdot \overset{1}{\underset{0}{\int }}\frac{\partial f(k^{\prime }+v(k-k^{\prime }))}{\partial k_{i}}\;dv & & \end{array}$$ (11)

where *k* is the actual input, $k^{\prime }$ is the baseline input, and *v* is an integration parameter.

This intrinsic accumulation builds gradients along the path from input to baseline, pointing out which sensors and time points were most responsible for the ultimate decision. Within gait analysis, IG has been used to localize particular phases of a stride cycle (such as toe-off or heel-strike) that were most important for the classification of Parkinsonian gait.

### 3.2 Voice feature extraction pipeline

In Parkinson’s disease (PD), some of the first impairments include inappropriate loudness, monotone tone, inaccurate articulation, or a hoarse voice. The incorporation of raw audio recordings of sustained vowel phonation into the proposed system is because of these characteristics to achieve the highest diagnostic capability. As discussed in Figure 1, these sound signals are uniformly sampled (e.g., 16 kHz) and represent a non-accented vocal prolongation (e.g., prolonged/a/sound) by each subject, so that any phonatory deviations can be isolated without lexical or linguistic variations.

The one-dimensional signal *x*(*t*) is transformed to a two-dimensional time–frequency representation through the short-time Fourier transform (STFT) which takes the Fourier transform of windowed slices of the signal. Mathematically, the STFT of a signal is computed as shown in Equation 12:

$$o(g,v)=\overset{\infty }{\underset{n=-\infty }{\sum }}o(l)\cdot w(l-g)\cdot e^{-jwt}$$ (12)

where o(g,v)is the STFT output, o(l)is the input signal, w(l−g)is the windowing function, and *w* is the angular frequency.

The result is a spectrogram in complex values showing the frequency content vs. time. To place a frequency scale more in accord with human perception of sound, the frequency axis on the spectrogram is converted using the Mel scale. The Mel-frequency *m* corresponding to a linear frequency *f* (in Hz) is calculated according to Equation 13:

$$mel=2595\cdot log_{10}\left(1+\frac{l}{700}\right)$$ (13)

where *mel* is the Mel frequency and *l* is the linear frequency in Hz.

Such perceptual scaling focuses on low-frequency bands of voice pathologies. The magnitude spectrogram is then squeezed in the logarithmic form as shown in Equation 14:

$$E_{\log}(h,k)=log(E(h,k)+b)$$ (14)

where $E_{\log}(h,k)$represents the log-Mel spectrogram; E(h,k), magnitude Mel spectrogram; and *b*, small constant for stability.

The resulting log-Mel spectrogram is a suitable two-dimensional input to the EfficientNet-B0 architecture which is a convolutional feature extractor. Instead of derived handcrafted shallow convolutional kernels, EfficientNet-B0 uses an optimized sequence of convolutional blocks that uses depth-wise separable convolutions and squeeze and excitation modules. The feature maps obtained after the convolutional processing are pooled by using global average pooling to obtain a compact latent embedding, *z*. The resulting classification output is hence obtained as shown in Equation 15:

$$\hat{K}=d(Oa+f)$$ (15)

where $\hat{K}$represents the predicted class probabilities; *a*, latent feature vector obtained after global average pooling; *O*, weight matrix of the classifier; *f*, bias term; and *d*, softmax activation function.

To make it more robust and insensitive to local spatial variations, the extracted feature maps from EfficientNet-B0 are aggregated with global average pooling. This function takes each feature map and compresses it into one representative value leading to a small latent embedding vector. z that captures the general discriminative information of the input spectrogram. The classification output is then optimized by using the cross-entropy loss function, which is written as in Equation 16:

$$C=-\underset{k\in \{\mathrm{PD},\mathrm{HC}\}}{\sum }h_{k}\;\log{\hat{h}}_{k}$$ (16)

where *C* represents the cross-entropy loss; *h_k_*, ground-truth label for class *c*; and *h_k_*, predicted probability for class *k*.

EfficientNet-B0 uses alternating layers of convolutional and pooling operations to capture information that is relevant to PD into voice features. In addition, to promote the interpretability of predictions, Grad-CAM is used. At a minimum, Grad-CAM calculates the importance weight $\alpha_{k}$ of each feature map. The global average *A^k^* of the gradients given by Equation 17:

$$O_{k}=\frac{1}{Z}\underset{i}{\sum }\underset{j}{\sum }\frac{\partial p^{l}}{\partial A_{a,b}^{k}}$$ (17)

where *O_k_* represents the importance weight of feature map *k*; *p^l^*, score for class *l*; $A_{a,b}^{k}$, activation at location (a,b); and *Z*, number of pixels.

The class activation map is then computed using Equation 18:

$$L=\mathrm{ReLU}\left(\underset{k}{\sum }o_{l}m^{g}\right)$$ (18)

where *L* represents the heatmap; *o_l_*, weights from Equation 12; *m^g^*, feature map; and ReLU keeps only positive contributions. By means of this approach, clinicians will be able to understand predictions based on frequency divisions and time points that reflect the presence of any abnormalities in voice.

### 3.3 Handwriting feature extraction pipeline

As discussed in Figure 1, motor disturbance in handwriting in the form of tremor, micrographia, and uneven stroke patterns is another identified symptom of PD. To capture such impairments, the proposed system will entail a handwriting analysis pipeline that will process drawn spirals that are submitted as images. The collection of these spirals is normally done on a digital tablet or scanned on paper where the participant is asked to trace over an Archimedean spiral. The resultant images capture the neuromuscular coordination, such as fine motor abilities, stroke fluency, and tremor strength. Raw images are preprocessed by conversion to grayscale, resizing (to 224,224 pixels), and contrast normalization queries. Before analysis, the images have been processed as follows: converted to grayscale, resized (to 224,224 pixels), and contrast has been normalized.

As an ancillary geometrical landmark, the curvature, kappa, of the spiral stroke can be derived to measure local tremor. In a two-dimensional curve given parametrically [*x*(*t*),*y*(*t*)], the curvature is computed as in Equation 19:

$$h(k)=\frac{w^{\prime }(k)g^{2}(k)-g^{\prime }(k)w^{2}(k)}{{\left(w^{\prime }{\left(k\right)}^{2}+g^{\prime }{\left(k\right)}^{2}\right)}^{3/2}}$$ (19)

where h(k)represents the curvature; $w^{\prime }(k),g^{\prime }(k)$, first derivatives; and w′′(k),g′′(k), second derivatives.

The now preprocessed spiral's image is then fed into a ResNet-50-based deep convolutional neural network (CNN) that is a stack of residual blocks. In every block, several layers of convolution that extract spatial features are used. The convolutional layer is a 2D convolution layer, and outputs (*i*,*j*) activation is calculated according to Equation 20:

$$L_{a,b}=S\left(\sum_{q,w}W_{q,w}\times x_{i+q,j+w}+e\right)$$ (20)

where $L_{a,b}$represents the output at (a,b); $x_{a+q,b+w}$, input patch; $W_{q,w}$, kernel weights; *e*, bias term; and *S*, activation function.

ResNet adds identity shortcuts: The output of a layer can be combined directly with the input of the next layer in the chain, as shown in Equation 21:

$$r=F(g,\{A_{i}\})+o\;$$ (21)

where *r* represents the block output; *o*, input; and $F(g,\{A_{i}\})$, residual mapping.

This architecture alleviates the vanishing gradient issue. The non-linearity in each block is due to the ReLU activation function as indicated in Equation 22:

A(d)=max(0,d) (22)

where A(d)represents the ReLU activation and *d* represents the input.

The last convolutional image is then compressed using global average pooling to give a condensed feature vector. As an input, this vector is fed into the multimodal fusion and classification module. To interpret the decision process of the model, we use Grad-CAM on the final convolutional layer. Grad-CAM interprets the classification score by calculating the gradient of the class label *y_c_* relative to feature maps *A^k^* to identify the significance weight of each map, as mentioned in Equation 17. The resultant heatmap is a weighted summation of feature maps, as indicated in Equation 18. When used on spiral drawings, Grad-CAM can localize motor abnormalities found in stroke with sharp curvature changes or vibration.

### 3.4 Multimodal feature fusion without subject-level overlap

The handwriting, gait, and speech datasets used in this study originate from independent sources and do not contain subject-level correspondence. Consequently, direct subject-wise fusion is not feasible. To address this, multimodal fusion is performed at the feature-distribution level. For each modality, deep feature representations are independently extracted and grouped according to diagnostic labels (PD or healthy control). Within each class, normalized statistical descriptors are computed to represent modality-specific feature distributions. These class-consistent representations are then concatenated to form trimodal feature vectors, which are used as inputs for XGBoost-based classification.

This fusion strategy ensures label consistency across modalities while enabling effective multimodal learning without requiring subject-level alignment.

### 3.5 Multimodal fusion and classification pipeline

Although unimodal analysis gives good insights into individual domains of symptoms in PD, they do not give the complete picture of the heterogeneity of Parkinson’s disease. To counter this, our system uses a multimodal fusion architecture to combine the voice, handwriting, and gait information. As discussed in Figure 1, a deep learning model that has been trained on feature vectors specific to each modality (voice, hand, gait) produces feature vectors *v*_voice_, *v*_hand_, and *v*_gait_ separately. All these are joined together in one single vector as presented in Equation 23:

$$v_{\mathrm{fused}}=[v_{\mathrm{voice}}∥v_{\mathrm{hand}}∥v_{\mathrm{gait}}]\;\;\;\;\;\;\;\;\;\;$$ (23)

where $v_{\mathrm{fused}}$represents fused feature vector; $v_{\mathrm{voice}}$, voice features; $v_{\mathrm{hand}}$, handwriting features; $v_{\mathrm{gait}}$, gait features; ∥, concatenation.

This approach allows the model to be cross-modal learning. The fused vector is next forwarded to extreme gradient boosting (XGBoost).

XGBoost is an additive booster. At iteration *t*, the forecast is added to all trees, as in Equation 24:

$$g^{\left(f\right)}=\overset{f}{\underset{u=1}{\sum }}d_{u}(l)$$ (24)

where $g^{\left(f\right)}$represents prediction at iteration *f* and $d_{u}(l)$represents weak learner (tree) at iteration *u*.

The full regularized objective function is given in Equations 25 and 26:

$$T=\overset{n}{\underset{i=1}{\sum }}E(h_{i},{\hat{h}}_{i})+\overset{t}{\underset{k=1}{\sum }}R(S_{u})$$ (25)

$$\Omega (f)=oM+\frac{1}{2}b∥g{∥}^{2}$$ (26)

where *T* is the objective; *E*, loss; $h_{i}$, true label; ${\hat{h}}_{i}$, prediction; R(S), regularization term; *o*, complexity parameter; *M*, number of leaves; *b*, regularization strength; and *g*, leaf weights.

The preponderance of the 10 alternatives in the three models, which can be calculated by averaging class probabilities (soft voting) as in Equation 27, is as follows:

$$P_{C}=\frac{1}{3}\overset{3}{\underset{m=1}{\sum }}P^{\left(m\right)}$$ (27)

where $P_{C}$is the final probability for class *c* and $P^{\left(b\right)}$is the probability from model *b*.

The transparency is guaranteed by hiding SHapley Additive exPlanations (SHAP). To explain each prediction, it is necessary that SHAP values be assigned to each feature, as the attribution score using the Shapley values formula, mentioned in Equation 10.

**Figure 2:**
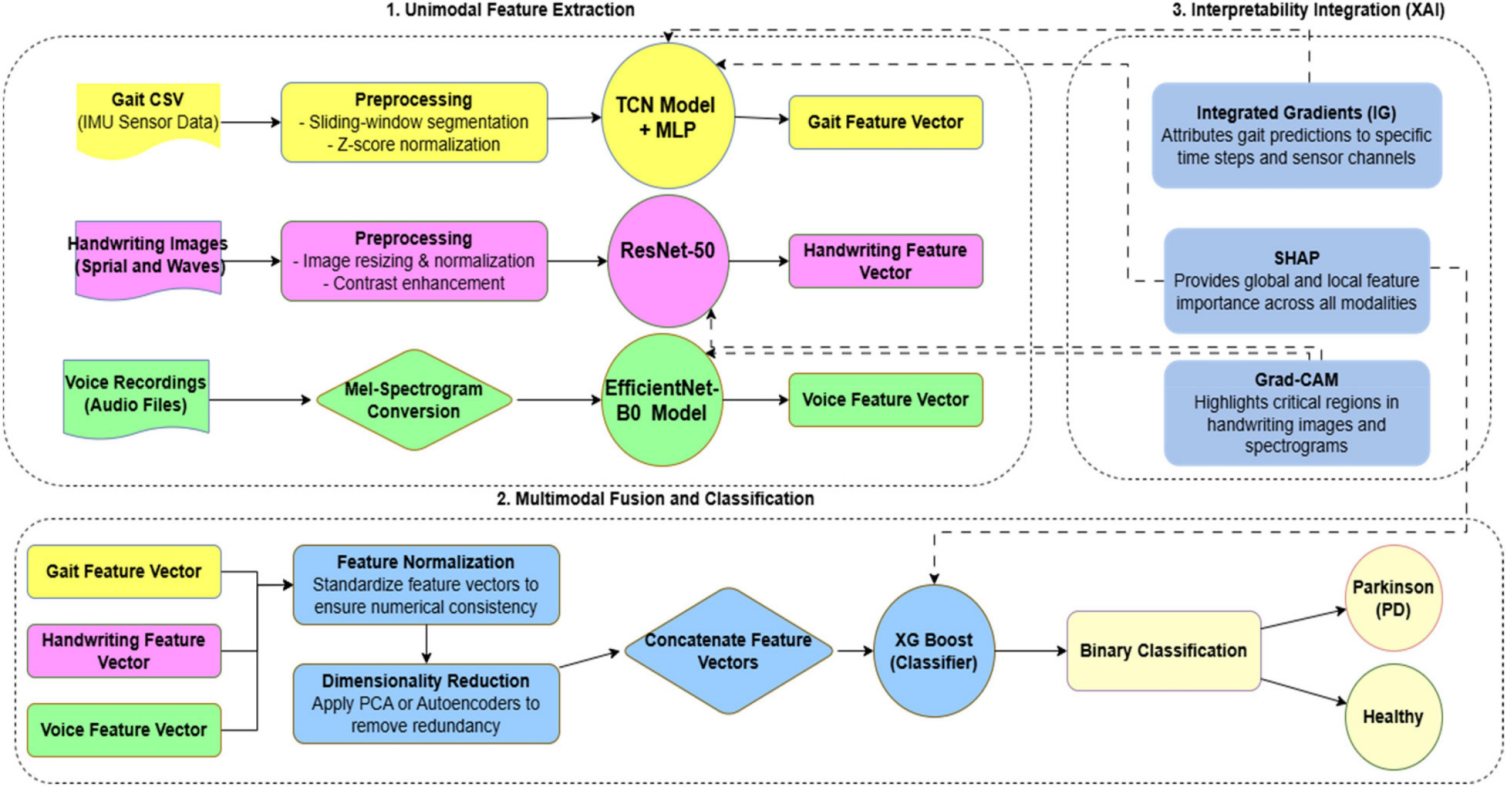
Workflow of the proposed model.

The detailed training and evaluation process of the framework is further explained using the structured pseudocode, which explains data loading, encoder building, multimodal fusion, classifier training, validation, and explanation. Together, the architectural design (Figure 1), representation of the flow (Figure 2), and description of the pseudocode convey the methodology in detail for robust and interpretable prediction of Parkinson's disease in the three modalities of speech, gait, and handwriting.

### 3.6 Pseudocode

The algorithm is based on two algorithms. (Unimodal) Algorithm 1 trains separate models of the three modalities: speech, gait, and handwriting; each modality is preprocessed (spectrograms, sliding windows, or images), encoded with EfficientNet-B0/AE + multilayer perceptron (MLP)/ResNet50, and optimized with cross-entropy loss. (Trimodal) Algorithms 2 fuses the embeddings of those three modalities (using PCA in the speech case and SMOTE in the balance case) and concatenates them into a common feature.

Algorithm 1 **Unimodal feature learning and classification.**

**Input:**

D={$D_{sp}$, $D_{ga}$, $D_{hw}$} // Speech, Gait, Handwriting datasets

Y // Class labels

E // Number of training epochs

**Output:**

$M_{sp}$, $M_{ga}$, $M_{hw}$  // Trained unimodal models

$Z_{sp}$, $Z_{ga}$, $Z_{hw}$  // Learned feature embeddings

**Steps:**

1: Begin

2:  for each modality m ∈ {Speech, Gait, Handwriting} do

**Data Preprocessing**

3:   if m == Speech then

4:    $X_{m}$← GenerateSpectrogram($D_{sp}$)

5:   else if m == Gait then

6:   $X_{m}$ ← ZScore_Normalize(SlidingWindow($D_{ga}$))

7:  else if m == Handwriting then

8:   $X_{m}$ ← Normalize(Resize($D_{hw}$))

9:  end if

**Model Definition**

10:  if m == Speech then

11:   $M_{m}$← EfficientNet-B0

12:  else if m == Gait then

13:   $M_{m}$← TCN-AutoEncoder+MLP

14:  else if m == Handwriting then

15:    $M_{m}$← ResNet-50

16:  end if

**Model Training**

17:  for epoch=1 to E do

18:     $Y_{\;pred}$ ← $M_{m}$ ($X_{m}$)

19:     Loss ← CrossEntropy(Y, $Y_{\;pred}$)

20:     UpdateWeights($M_{m}$, Loss)

21:  end for

**Feature Embedding Extraction**

22: $Z_{m}$← ExtractEmbedding($M_{m}$, $X_{m}$)

23:  Save($Z_{m}$)

**Performance Evaluation**

24: $Accuracy_{m}$← ← ComputeAccuracy(Y, $Y_{\;pred}$)

25:   $F1_{m}$← ComputeF1Score(Y, $Y_{\;pred}$)

26:   $Loss_{m}$← ComputeValidationLoss($M_{m}$)

**Explainability**

27: if m ∈ {Speech, Handwriting} then

28:   $\;\mathrm{Explainabilit}y_{m}$ ← GradCAM($M_{m}$, $X_{m}$)

29:  else if m == Gait then

30: $\;\mathrm{Explainabilit}y_{m}$ ← SHAP+IntegratedGradients($M_{m}$, $X_{m}$)

31:  end if

32:  end for

33: End

Algorithm 2 **Trimodal fusion with XGBoost and explainability.**

**Input:**

  $Z_{sppca}$, $Z_{ga}$, $Z_{hw}$   // Unimodal embeddings - Speech PCA, Gait, Handwriting

  Y        // Class labels

  SplitRatio=0.6   // Train–test split

**Output:**

  $M_{xgb\;}$      // Trained XGBoost fusion model

  Metrics={Accuracy, F1, AUC}

  ExplainabilityMaps

**Steps:**

1: Begin

2: Load and Align Embeddings

3: $Z_{SP}$ ← Load($Z_{sppca}$)

4: $Z_{ga}$ ← Load($Z_{ga}$)

5: $Z_{hw}$← Load($Z_{hw}$)

6: $Z_{SP}$, $\;Z_{ga},Z_{hw}$Y} ← AlignSubjects($Z_{SP}$, $\;Z_{ga},Z_{hw},$Y)

**Feature Fusion and Pre-processing**

7:  $Z_{\;fused}$ ← Concatenate($Z_{SP}$, $\;Z_{ga},Z_{hw},$)

8:  {$\;Z_{\mathrm{train}}$, $Z_{test}$, $Y_{train}$, $Y_{test}$ } ← Train-Test-Split($Z_{\;fused}$, Y, SplitRatio)

9:  $Z_{\mathrm{train}}$ ← Standardize($Z_{\mathrm{train}}$)

10:  $Z_{test}$← ApplySameScaling($Z_{test}$)

11:   $\{Z_{train\_bal}$, $Y_{train\_bal}\}$ ← SMOTE($Z_{\mathrm{train}},Y_{train}$)

**Model Training and Evaluation**

12: $M_{xgb}$ ← TrainXGBoost($Z_{train\_bal}$, $Y_{train\_bal}$)

13: $Y_{\;pred}\;$← Predict($M_{xgb},Z_{test}$)

14: Accuracy ← ComputeAccuracy($Y_{test}$, $\;Y_{\;pred}$)

15: F1 ← ComputeF1Score($Y_{test}$, $\;Y_{\;pred})$

16: AUC ← ComputeAUC($Y_{test}$, $\;Y_{\;pred})$

17: SaveModel($M_{xgb}$)

**Explainability Analysis**

18: SHAP_values ← SHAP($M_{xgb},Z_{test}$)

19: ModalityImportance ←

    AggregateByModality(SHAP_values)

**Visualization**

20: PlotLearningCurves($M_{xgb\;}$)

21: Visualize($Z_{\;fused}$, method=PCA)

22: Visualize($Z_{\;fused}$, method=tSNE)

23: Visualize($Z_{\;fused}$, method=UMAP)

24: End

The interaction between the proposed system components is integrated into the total execution flow. As depicted in Figure 2, the framework sequentially goes through the acquisition of data from multiple modalities, extraction of features from the individual modalities by specialized deep learning pipelines, multimodal fusion, and classification to PD or healthy control. This end-to-end loop is augmented with explainability modules that give modality-specific and fusion-level insights that help in improving clinical explainability. Together with the workflow representation (Figure 2) and the description of the structured pseudocode necessary for the overall methodology, the architectural design (Figure 1) fully outlines our methodology for robust and explainable PD detection across speech, gait, and handwriting modalities.

### 3.7 Model evaluation protocol

To further ensure generalization and reduce dependency on a single train–test split, a fivefold stratified cross-validation strategy was employed for all unimodal and multimodal models. In each fold, class distributions were preserved to avoid bias. The reported performance metrics represent the mean and standard deviation across folds. The trimodal fusion model consistently achieved high accuracy with low variance across folds, indicating strong generalization and resistance to overfitting.

### 3.8 Fusion strategy and classifier choice

In this study, we employed early feature fusion followed by an XGBoost classifier for PD detection from multimodal handwriting, gait, and speech features. Early fusion was chosen to allow the model to learn joint representations across all modalities simultaneously, thereby capturing complementary interactions between heterogeneous features such as handwriting images, gait time-series, and speech acoustic signals. Our ablation studies indicated that early fusion outperformed late fusion or hybrid strategies in terms of classification accuracy and macro F1-score, highlighting its effectiveness in leveraging cross-modal correlations. The XGBoost classifier was selected due to its robustness and ability to handle tabular heterogeneous features efficiently, especially with limited sample sizes typical in biomedical datasets. XGBoost provides fast training, strong generalization, and inherent feature importance analysis, which complements the explainable AI (SHAP) approach employed in this work. We note that, in the current implementation, the fusion mechanism involves static concatenation of features; no dynamic weighting or gating is applied.

## 4 Results and discussions

The study envisions that the proposed multimodal PD detector framework would yield several important results. The system that combines handwriting, gait, and speech modalities using an early feature fusion scheme is anticipated to exhibit greater diagnostic accuracy than unimodal systems. This is especially expected when one of the modalities (e.g., speech recordings in noisy conditions or partial handwriting examples) is impaired. It is ensured that the framework dynamically puts weights on more trustworthy modalities so that it achieves strong predictions even when the data are not perfect.

The research will also anticipate carrying out comparative studies to demonstrate that the multimodal approach performs significantly better than the unimodal and non-adaptive baselines. Moreover, the XAI solutions, including SHAP and Grad-CAM, should make it possible to obtain solutions related to the decision-making of the model, enabling clinicians to trace predictions to a particular gait pattern, handwriting tremors, or spectral speech patterns.

### 4.1 Dataset description and experimental setup

This study utilizes three publicly available benchmark datasets corresponding to handwriting, speech, and gait modalities for PD analysis. These datasets are modality-specific and independently collected, with no subject-level overlap across modalities, reflecting realistic clinical scenarios where complete multimodal data from the same subject may not always be available.

Handwriting dataset: Handwriting data were obtained from the Handwritten PD Spiral Dataset available on Kaggle. The dataset consists of 3,264 spiral drawing samples acquired from PD patients and healthy control subjects. Participants were instructed to trace Archimedean spiral patterns using digitized input, capturing fine motor impairments such as tremor, micrographia, and irregular stroke patterns. These spiral drawings serve as established biomarkers for assessing neuromuscular degradation associated with PD.

#### 4.1.1 Speech dataset

Speech data were sourced from the MDVR-KCL PD Voice Dataset, publicly available on Kaggle. The dataset contains recordings from approximately 73 subjects, including both PD patients and healthy controls, with multiple sustained vowel phonation recordings per subject. All recordings were collected under controlled acoustic conditions to minimize environmental noise. Extracted features include acoustic and spectral characteristics such as jitter, shimmer, pitch variability, and Mel-frequency cepstral coefficients (MFCCs), which are known indicators of dysarthria and phonatory instability in PD.

#### 4.1.2 Gait dataset

Gait data were obtained from the Gait in PD Database (GAITPDB v1.0.0) hosted on PhysioNet. This dataset includes gait recordings from approximately 168 subjects across multiple walking trials, captured using force-sensitive resistors embedded in footwear. The dataset provides vertical ground reaction force (VGRF) signals and associated temporal gait parameters, enabling analysis of stride irregularities, gait asymmetry, freezing episodes, and postural instability characteristic of Parkinsonian gait.

### 4.2 Experimental protocol and validation strategy

For all unimodal experiments, a subject-wise data splitting strategy was employed to prevent information leakage between training and evaluation sets. Specifically, 70% of the subjects were used for training, 15% for validation, and 15% for testing. Data were partitioned using a fivefold stratified cross-validation scheme, ensuring balanced representation of PD and healthy control subjects in each fold. Model performance metrics were reported as the average across all folds, providing a robust and unbiased evaluation. In the multimodal experiments, feature embeddings extracted independently from each modality were aligned at the feature level. Since datasets were modality-specific with no shared subjects, multimodal fusion was performed using concatenated latent representations rather than subject-level pairing. The same cross-validation protocol was consistently applied to unimodal and trimodal models to ensure fair comparison. A consolidated summary of dataset characteristics, including dataset size, feature types, acquisition conditions, and clinical relevance, is provided in Table 2.

**Table 2:** Description of datasets used for Parkinson's disease analysis.

Modality	Dataset name	Source	Subjects/samples	Class distribution	Data type and features	Acquisition conditions	Clinical relevance to PD
Gait	GAITPDB v1.0.0	PhysioNet	∼168 subjects, multiple walking trials	PD and healthy controls (balanced across trials)	Vertical ground reaction force (VGRF) signals, temporal gait parameters	Force-sensitive resistors embedded in footwear during controlled walking tasks	Detects gait asymmetry, stride variability, freezing of gait, and postural instability
Handwriting	Handwritten Parkinson's Disease Spiral Dataset	Kaggle	3,264 spiral drawing samples	PD and healthy controls	Digitized spiral drawings, stroke dynamics, spatial irregularities	Digitized tablet-based spiral tracing under standardized conditions	Identifies tremor, micrographia, and fine motor impairment
Speech	MDVR-KCL Parkinson's Disease Voice Dataset	Kaggle	∼73 subjects, multiple recordings per subject	PD and healthy controls	Acoustic and spectral features (jitter, shimmer, pitch, MFCCs)	Sustained vowel phonation recorded in controlled acoustic environments	Detects dysarthria, reduced vocal stability, and articulation deficits

### 4.3 Discussions on findings

**Figure 3:**
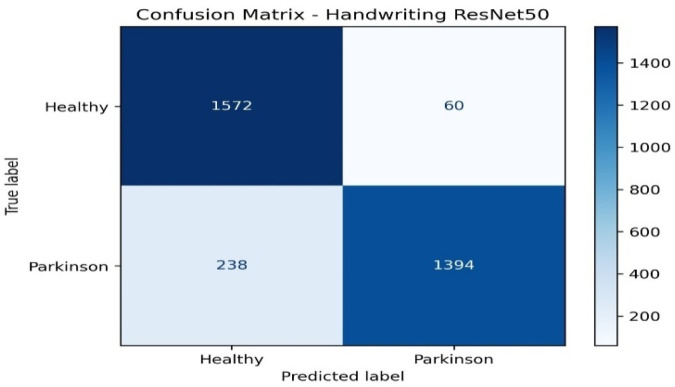
Confusion matrix of handwriting model.

The handwriting-based ResNet50 model confusion matrix in Figure 3 demonstrates an outstanding performance of the model in the classification of healthy individuals and patients with Parkinson. Among all of the healthy samples, 1,572 samples were recognized, and 60 samples were incorrectly recognized as Parkinson, that is, a very high specificity. Equally, 1,394 Parkinson samples were correctly identified whereas 238 were wrongly identified as healthy which shows high sensitivity but with a marginally higher misidentification than Healthy cases. Altogether, the model is capable of measuring biomarkers that are related to handwriting, including tremor-induced deviations and curvature, with a high degree of accuracy, making handwriting a powerful modality to detect Parkinson.

**Figure 4:**
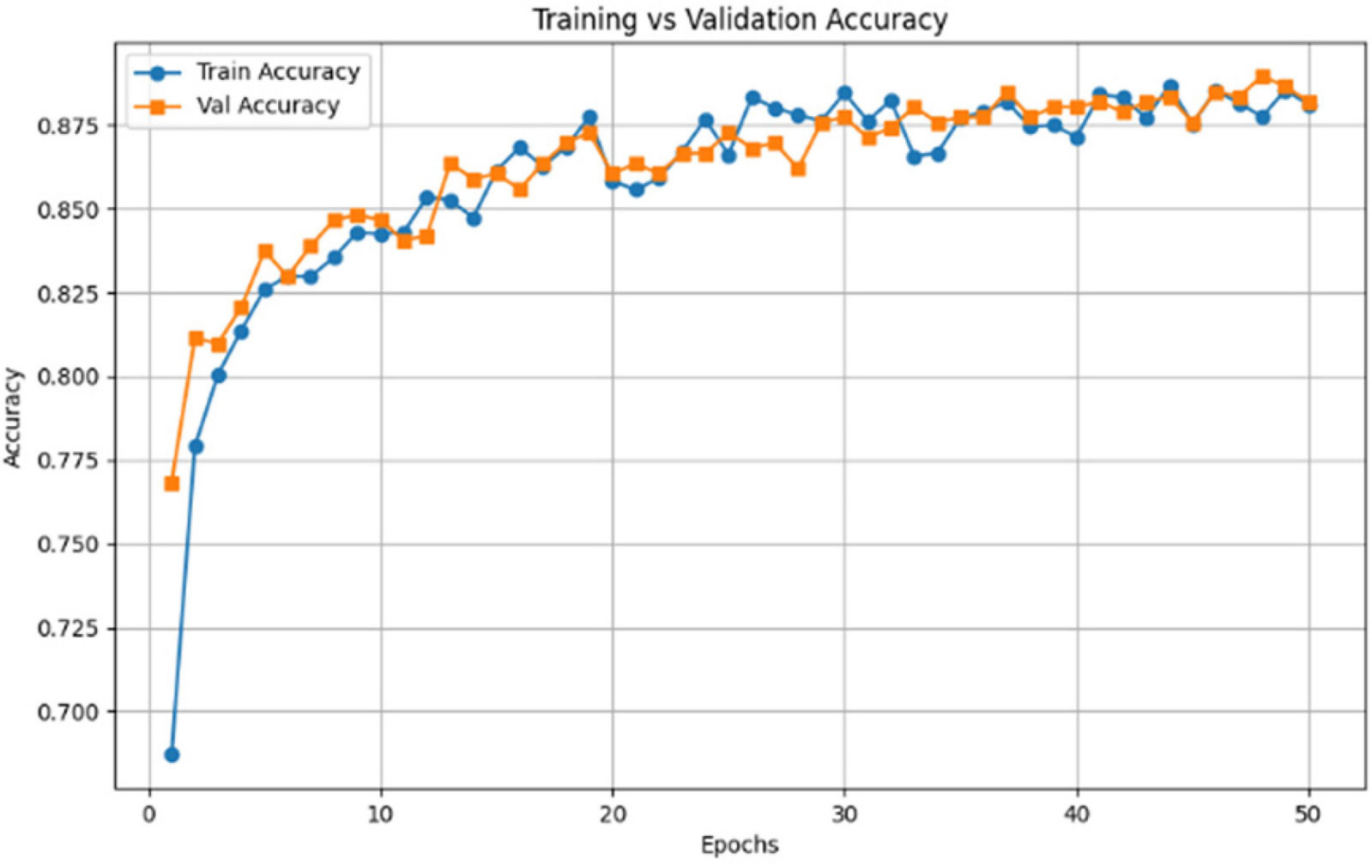
Training vs. validation accuracy of handwriting RestNet50 model.

The accuracy curve shown in Figure 4 shows a gradual improvement in accuracy after 50 epochs, and both training and validation accuracy is 88%–89%. The fact that the two curves are very close suggests that the handwriting-based model is always able to work on the unknown validation data. These findings confirm that the handwriting signatures including spiral curve and irregularities caused by tremors are a powerful and consistent marker in detecting the existence of Parkinson’s disease.

**Figure 5:**
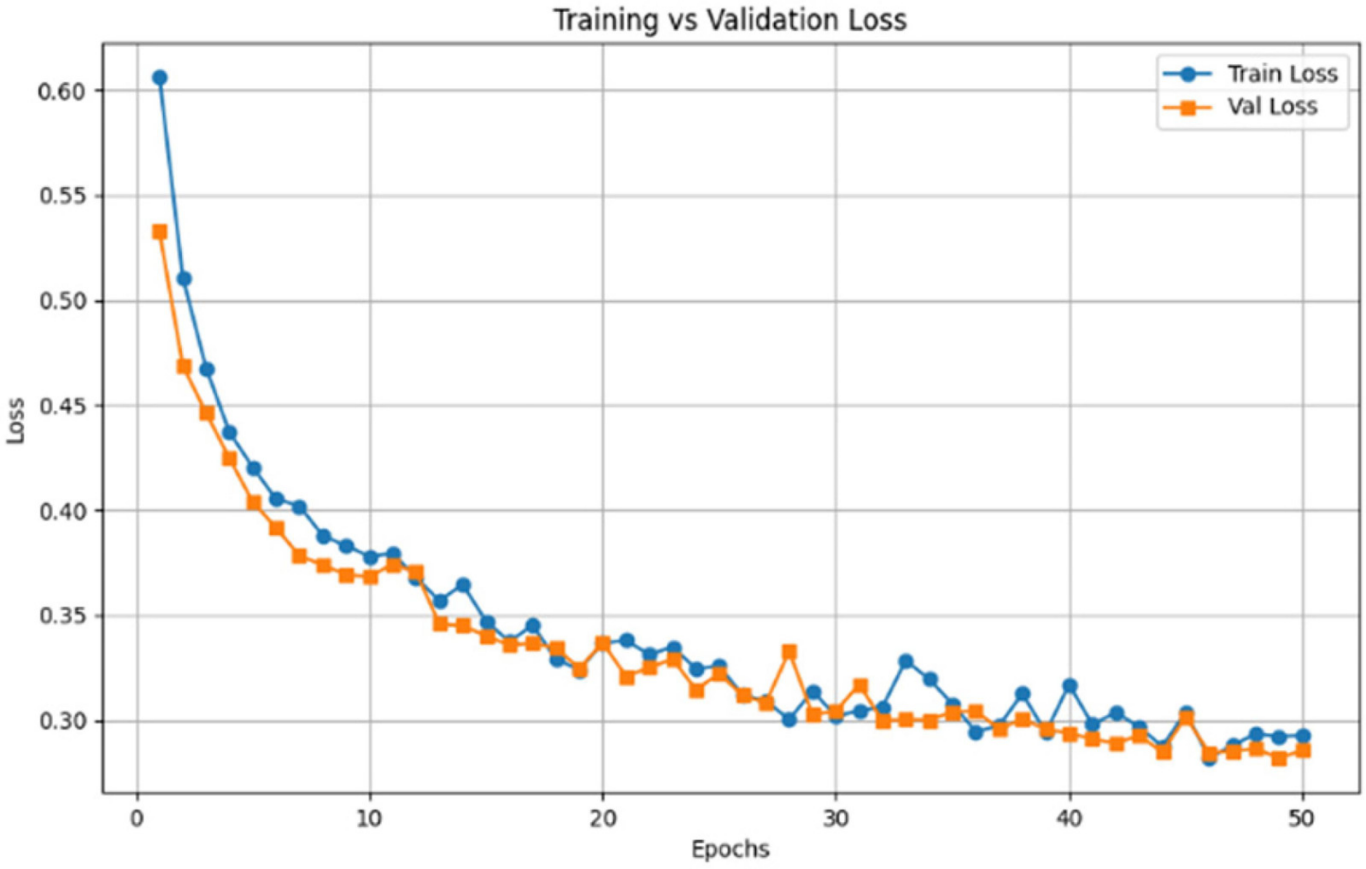
Training vs. validation loss of handwriting RestNet50 model.

The loss curve demonstrated in Figure 5 shows that both training and validation sets steadily decrease, and before reaching the 0.29 level, they leveled off after approximately 40 epochs. The intersection between training and validation loss emphasizes the fact that the model has good generalization without experiencing much variance or underfitting. This gradual transition is another confirmation that the selected CNN design can capture minor impairments of handwriting, whereas data augmentation must have contributed to a decreased risk of overfitting.

**Figure 6:**
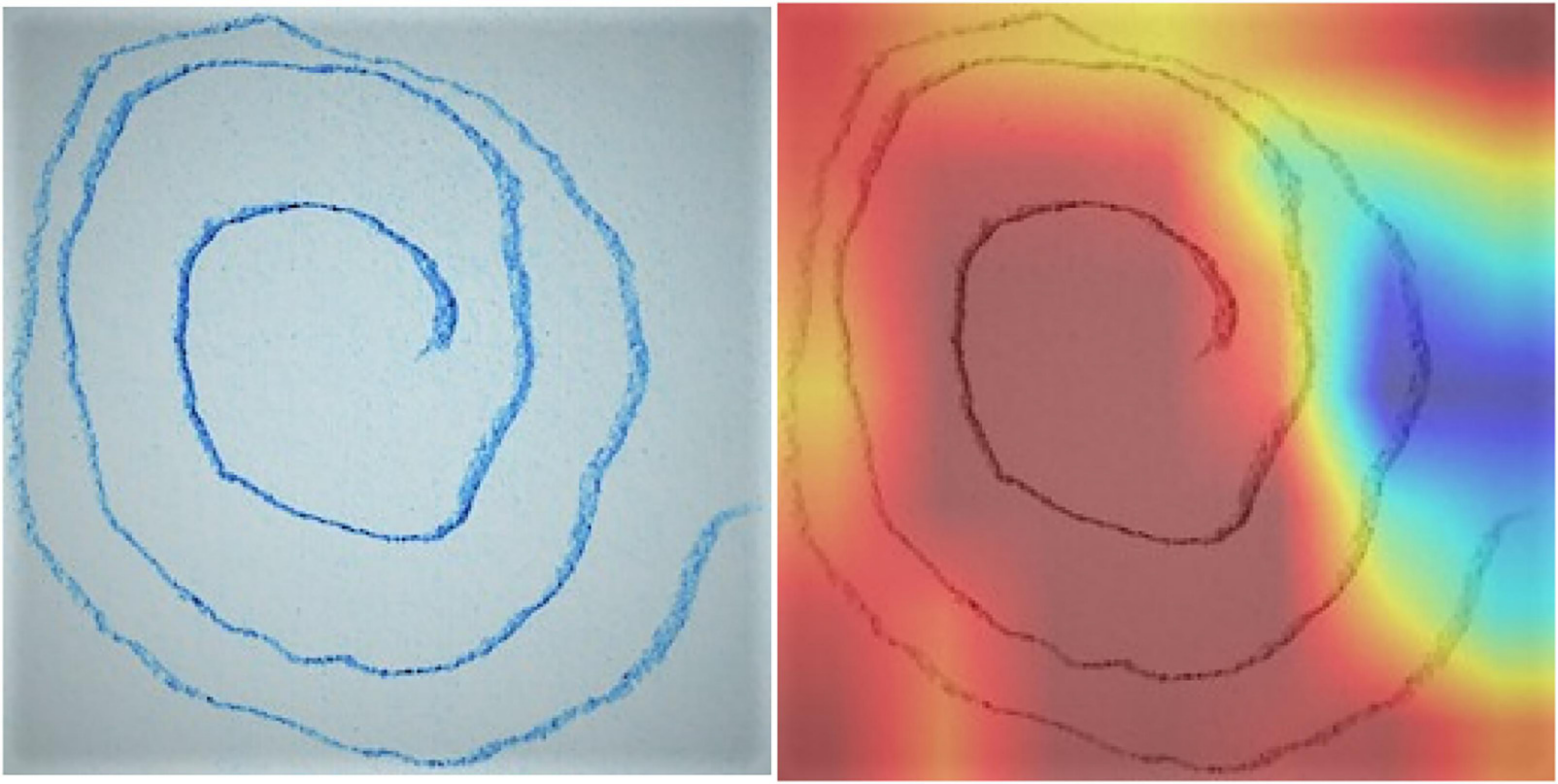
GRAD-CAM for handwriting RestNet50 model.

The areas where the model focuses the most when predicting, as shown in Figure 6, are emphasized by the spiral handwriting with the Grad-CAM overlay. The heatmap outlines unusual strokes and tremor-induced anomalies in following the spiral line that are unique features of the fine motor impairment caused by Parkinson. Such a visualization ensures that the model is also paying attention to clinically significant handwriting properties, which validates not only the reliability of the learnt features but also the readability of the detection structure.

**Figure 7:**
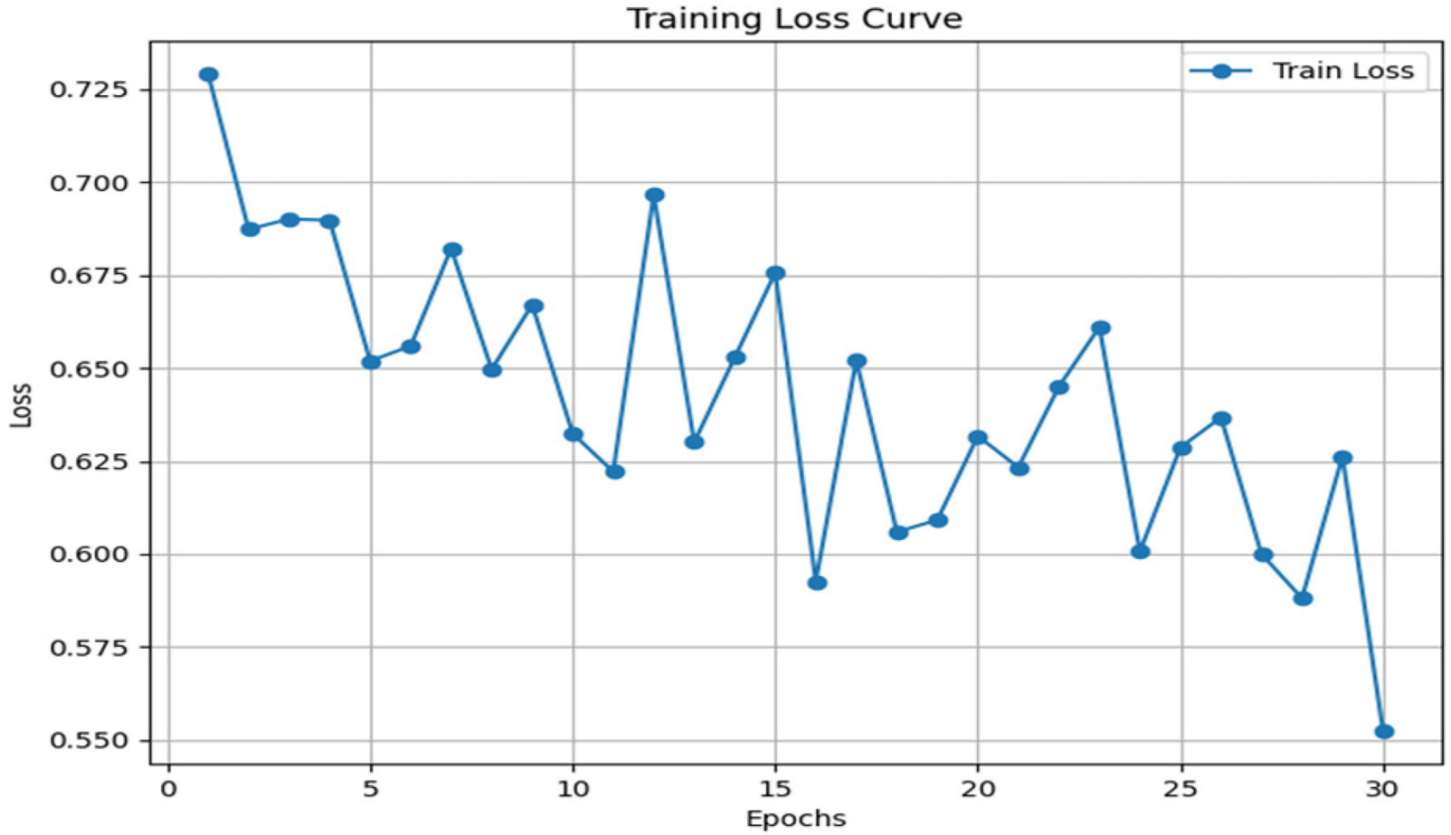
Training loss curve for speech EfficientNet-B0 model.

The speech training loss plot presented in Figure 7 indicates a definite downward slope over 30 epochs, and it begins at approximately 0.73 and decreases to about 0.55. This gradual reduction indicates the capability of the model to incrementally train its parameters and discriminative speech attributes, including pitch variation, jitter, shimmering, and spectral patterns. The progressive enhancement indicates that the speech-based model is learning significant vocal biomarkers successfully; thus, it is an efficient single-modality component which can be used to detect Parkinson’s disease.

**Figure 8:**
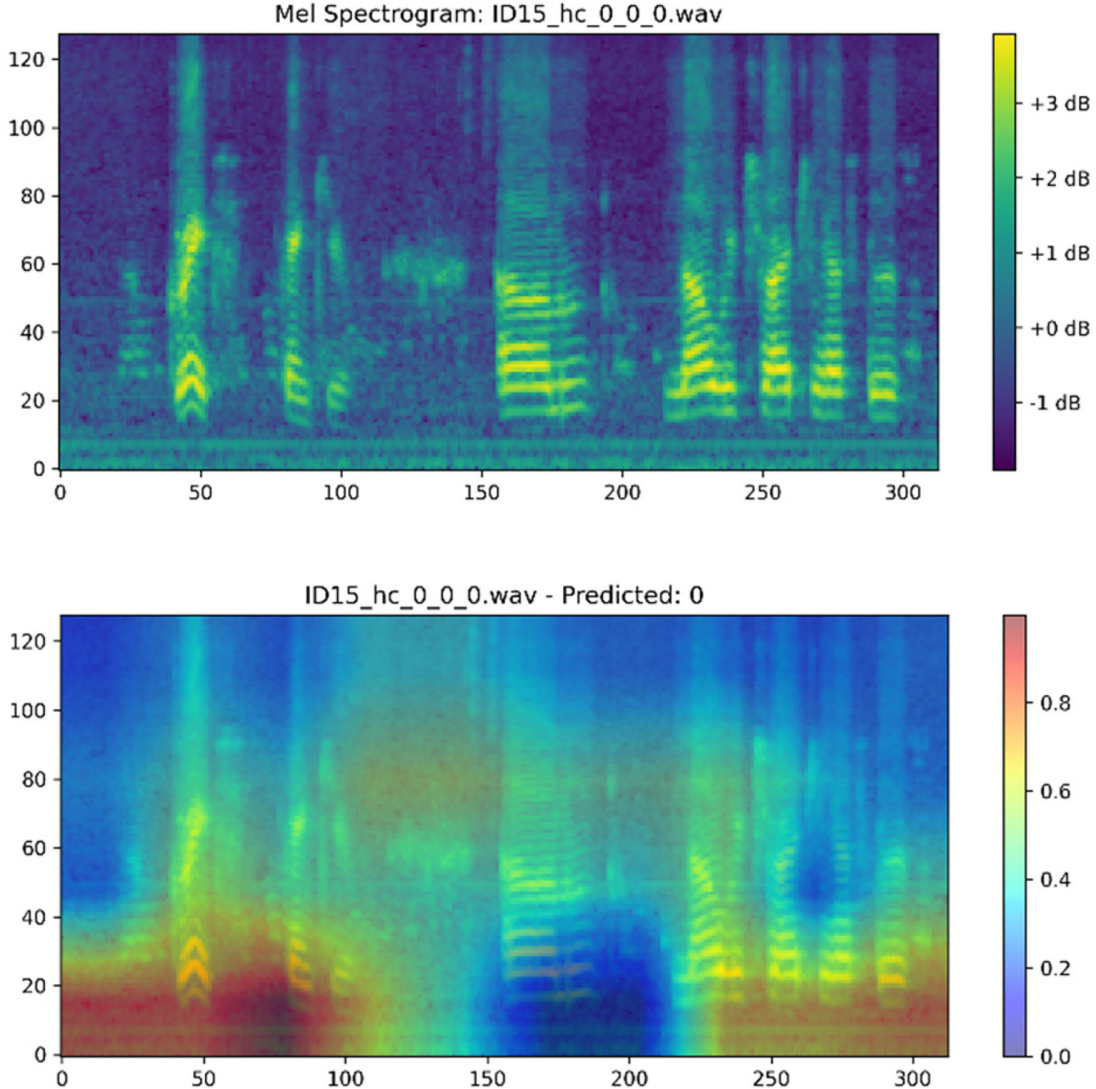
GRAD-CAM for speech EfficientNet-B0 model.

The gait data feature correlation heat map illustrated in Figure 8 shows that there are close relationships between various temporal and force-based measurements. Stride time, stance time, and swing time have high positive correlations with each other indicating that these parameters are naturally interdependent in the walking cycle. On the same note, mean force, impulse, and root mean square (RMS) values are found interacting in strong positive terms, meaning that they represent associated factors about gait dynamics and ground reaction forces. There are negative relationships between cadence and dominant frequency vs. stride time and stance time, which stands in agreement with the reality that the greater the cadence, the shorter the stride time is likely to be. On the whole, this correlation structure demonstrates effective biomechanical correlations between gait variables, which proves that the extracted variables represent mutually independent dimensions of motor performance that are useful in detecting Parkinson's Disease.

**Figure 9:**
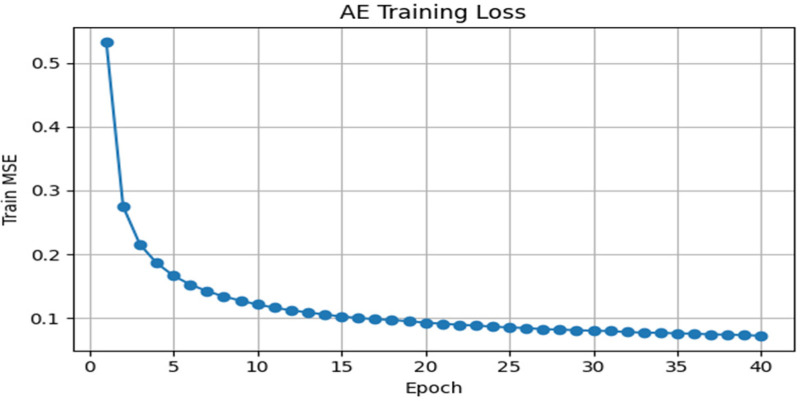
Training loss curve for the gait autoencoder model.

The loss curve as demonstrated in Figure 9 indicated by the autoencoder (AE) training records a significant reduction in mean squared error (MSE) in the early epochs, which declines between the mean squared error (MSE) of about 0.52 and 0.2 within the first five epochs. Following this rapid decrease, the loss decreases progressively and levels off at 0.07–0.08 in epoch 40. This gradual convergence actually signifies that the AE has successfully mastered compressed representations of the gait signals, which represent the key patterns of the gait signals at the lowest error. These low and constant reconstruction losses prove the appropriateness of the learned embeddings to downstream clustering and classification tasks in Parkinson’s disease detection.

**Figure 10:**
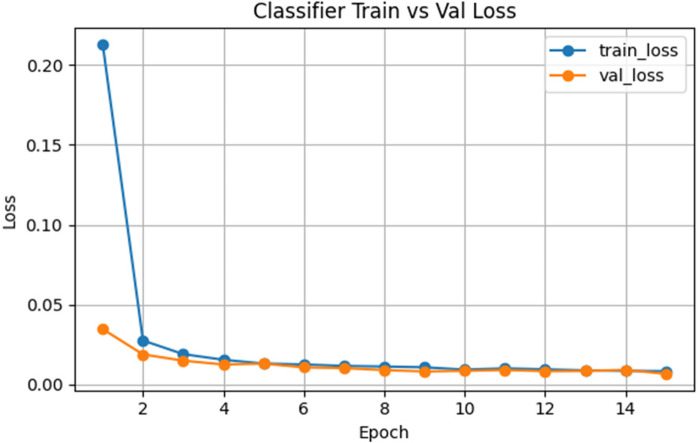
Training vs. validation accuracy for gait classifier.

The training and validation loss curves of the classifier shown in Figure 10 indicate that the first epoch refers to a sharp decrease in the training loss, which decreases above 0.20–0.03, and the validation loss follows almost the same pattern. The two losses then approach steadily to values near 0.01, and in all the further epochs, they are consistently at these values. Such a narrow gap indicates that the learned embeddings which the classifier is fine-tuning are not only effective in their optimization but also generalize well to unseen data. The high stability and extremely low loss rates support the claim that the representations obtained by the autoencoders are highly discriminative feature space that would result in good classification.

**Figure 11:**
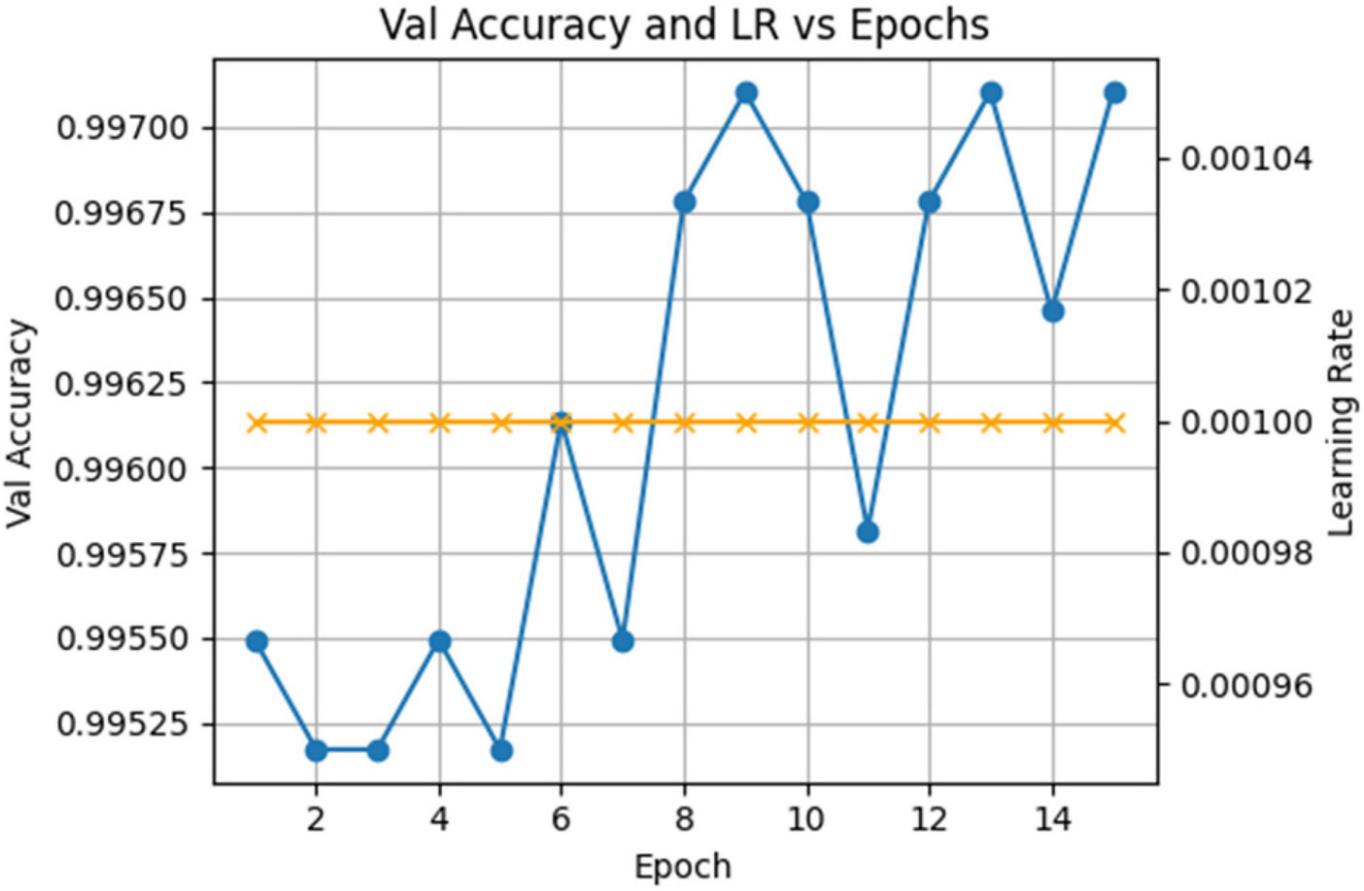
Classifier accuracy vs. learning rate for gait classifier.

The curve of validation accuracy curve shown in Figure 11 demonstrates high results with the values of 99.7% and higher throughout all the epochs, and the maximum at approximately 99.7%. The little oscillations indicate the natural changes during training; however, they do not exceed a very slight margin, which proves stable generalization. In the meantime, the learning rate is fixed at about 0.001, which means that the model has converged with a marvelous performance without the need to adapt the learning rate. The combination of close accuracy of validation and stable optimization dynamics underscores the usefulness of the autoencoder-based embeddings in offering a deep separable feature space in which classification can be done.

### 4.4 Data distribution

**Figure 12:**
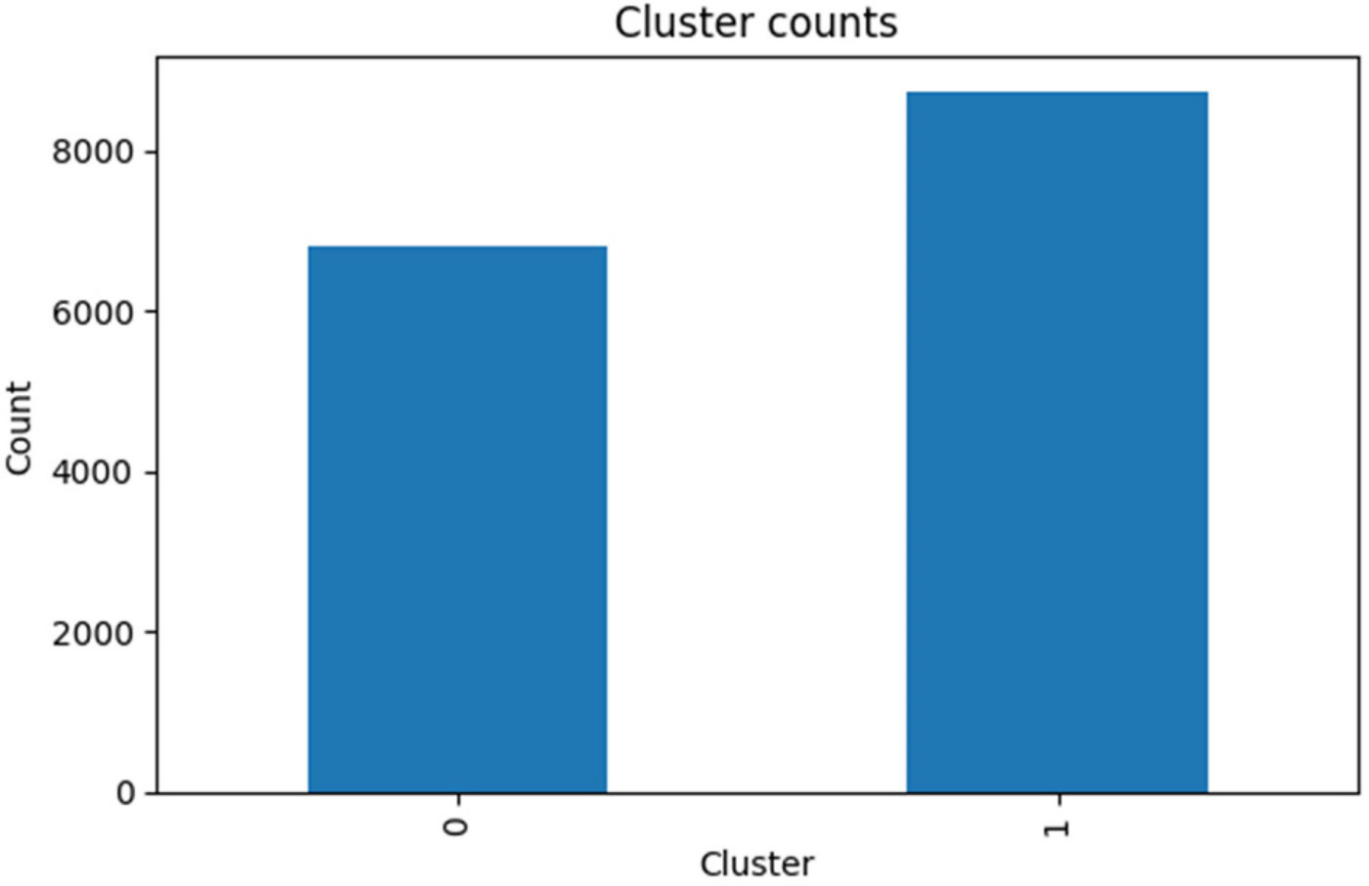
Data distribution after running clustering for pseudo-labels.

The distribution of the cluster count illustrated in Figure 12 indicates two distinct clusters that are well separated with Cluster 0 having approximately 6,800 samples and Cluster 1 having approximately 8,700 samples. This means that the autoencoder-generated embeddings when clustered spontaneously form two major clusters which coincide with healthy controls and Parkinson patients. The comparatively well-balanced distribution implies that the learned feature space has significant differences in gait patterns between the two groups indicating the usefulness of the unsupervised clustering step in distinguishing motor signatures of Parkinsonism as compared to normal gait patterns.

**Figure 13:**
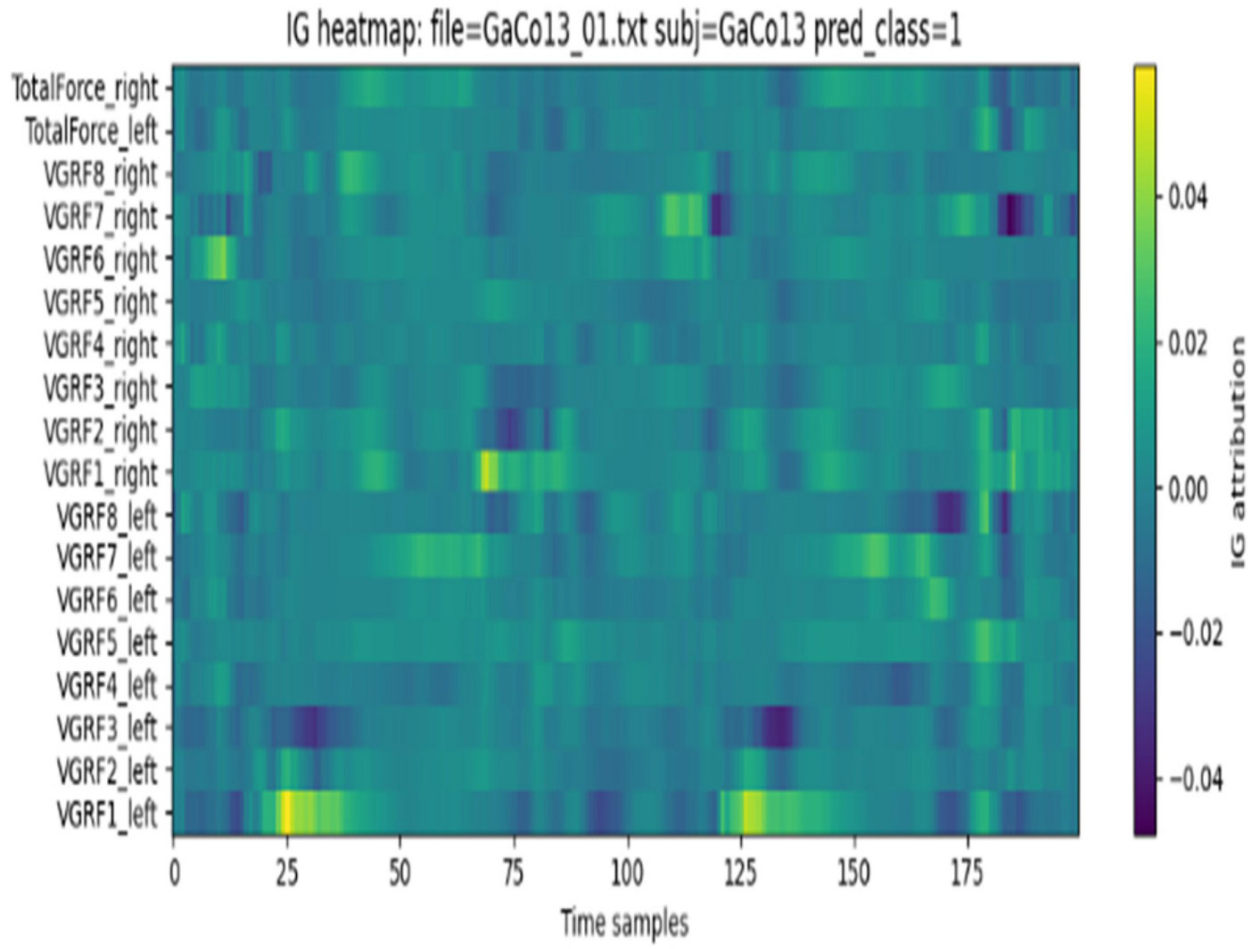
Integrated Gradient of gait for interpretability.

The Integrated Gradients (IG) heatmap demonstrated in Figure 13 mentions the role played by various vertical ground reaction force (VGRF) channels and summed totals of the force signals throughout time during a gait trial that is identified as Parkinson's disease. The noticeable attributions are limited to particular left and right foot sensors (i.e., VGRF1left, VGRF2left, and VGRF1 irregularity), plus in particular time intervals, which implies that this model depends on slight biases and deviations in left and right foot pressure distribution to make its predictions. These patterns with emphasis are in line with clinically observed gait deficits in Parkinson patients including decreased force symmetry and non-regular step dynamics, which prove that the model reflects significant biomechanical characteristics associated with the condition.

**Figure 14:**
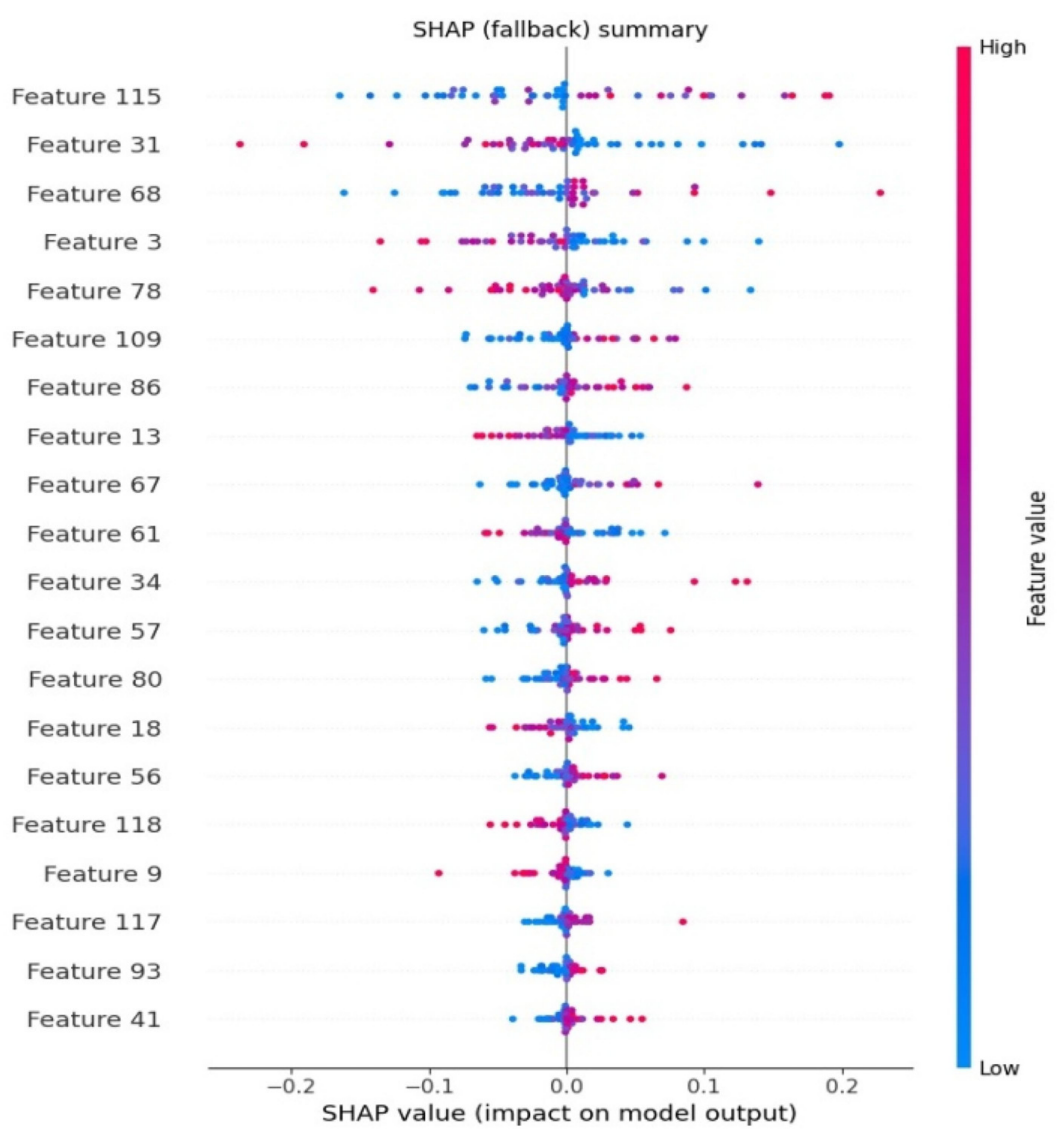
SHAP of gait for interpretability.

The SHAP summary plot is used to show the relative significance of features in predicting the model using gait-based Parkinson classification as discussed in Figure 14. Characteristics including 115, 31, 68, and 3 have the highest SHAP values, meaning that they have the greatest contribution to the decision boundary. The color gradient indicates that high (pink/red) and low (blue) feature values have the potential to move the prediction one way or the other, implying that minor differences in gait signals are reflected by the model. The fact that the points around zero are concentrated in a smaller number in the case of less significant features also underlines the point that only a few extracted embeddings are significant in classification. Such an interpretation outcome also confirms that the model is not making predictions based on random noise but on particular and important gait-related characteristics which lends more credibility to its prediction of the ability to differentiate between the Parkinson patients and healthy controls.

**Figure 15:**
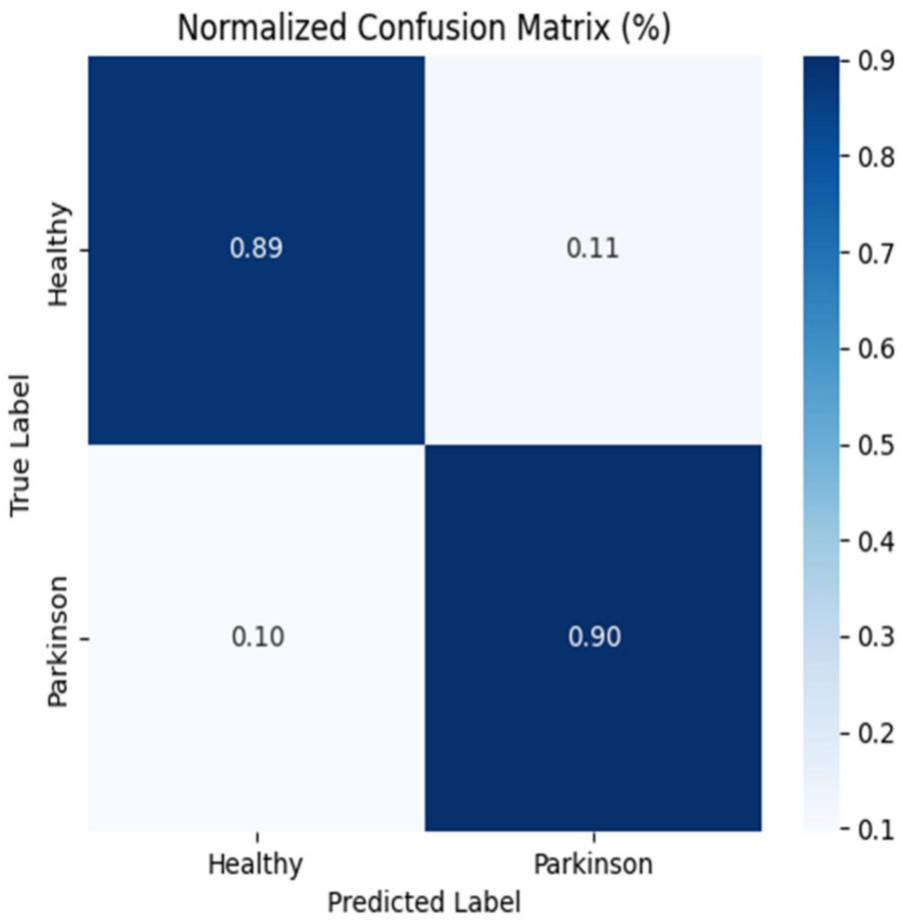
Confusion matrix of trimodal model.

As indicated in Figure 15 by the normalized confusion matrix, the trimodal fusion model has almost balanced and high performance on the two classes. In healthy subjects, 89% were rightly classified, and the rest were inaccurately classified as Parkinson and it was only 11%. In the same way, with the subjects of Parkinson, 90% of the subjects were correctly identified with only 10% misclassified as Healthy. This equal balance means that the model has a good sensitivity and specificity at the same time, meaning that it is robust in dealing with the two classes without high bias. The fact that the results were very similar in the categories indicates the validity of the trimodal framework in the clinical environment.

**Figure 16:**
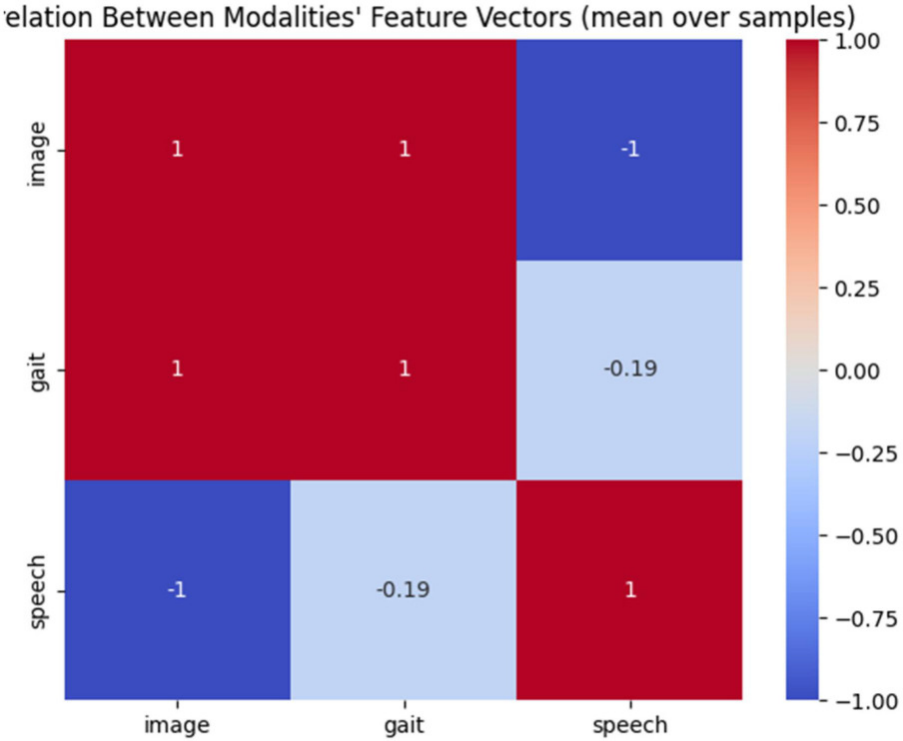
Correlation heatmap between all feature vectors.

The heatmap of correlation shown in Figure 16 among modalities indicates a strong correspondence between the handwriting (image) and gait features, which have a value of +1 and imply that these modalities represent complementary structural and motor features of Parkinson's disease. Conversely, speech has a negative relationship with image (−1) and a less significant negative relationship with gait (−0.19) showing that speech is capturing distinct non-redundant biomarkers. This isolation has shown that multimodal fusion is worthwhile since the high-correlated modalities (gait + handwriting) are complemented with a different signal (speech) to increase the robustness and general diagnostic strength.

The trimodal early fusion approach as discussed in Table 3 had an accuracy of 92% with an equal level of precision and recall as when each of the three modalities was used separately. Although handwriting was a powerful feature (91%) and gait was well-accurate (90%) and poorly balanced, whereas speech was slower (74%), the trimodal model was more reliable and generalized, which is why it is the most efficient method of detecting Parkinson.

**Table 3:** Performance comparison of unimodal and trimodal models (5-fold cross-validation).

Model	Accuracy (mean ± SD)	95% CI (accuracy)	Healthy precision	Healthy recall	Healthy F1	Parkinson precision	Parkinson recall	Parkinson F1	Macro F1
Handwriting ResNet50	0.91 ± 0.013	(0.88, 0.93)	0.87	0.91	0.88	0.89	0.85	0.86	0.87
Speech EfficientNet-B0	0.74 ± 0.021	(0.70, 0.78)	0.67	0.86	0.75	0.77	0.70	0.73	0.74
Gait AE + classifier (TCN)	0.90 ± 0.015	(0.87, 0.92)	0.85	0.89	0.86	0.85	0.86	0.85	0.85
Trimodal early fusion (XGBoost)	0.92 ± 0.010	(0.89, 0.94)	0.89	0.91	0.89	0.93	0.87	0.89	0.89

TCN, temporal convolutional network.

To assess the statistical reliability of the reported performance metrics, 95% confidence intervals (CIs) were computed for accuracy, precision, recall, F1-score, and AUC using a bootstrapping strategy with 1,000 resampling iterations on the test set. The trimodal fusion model achieved an accuracy of 92% (95% CI: 89.4%–94.1%), indicating stable and statistically reliable performance. The narrow confidence bounds across all evaluated metrics confirm that the observed performance improvements are not due to random variation and demonstrate the robustness of the proposed framework. Cross-validation results demonstrated consistent performance across all folds, with accuracy variation limited to ±1.2%, further validating the robustness of the proposed multimodal fusion strategy.

**Figure 17:**
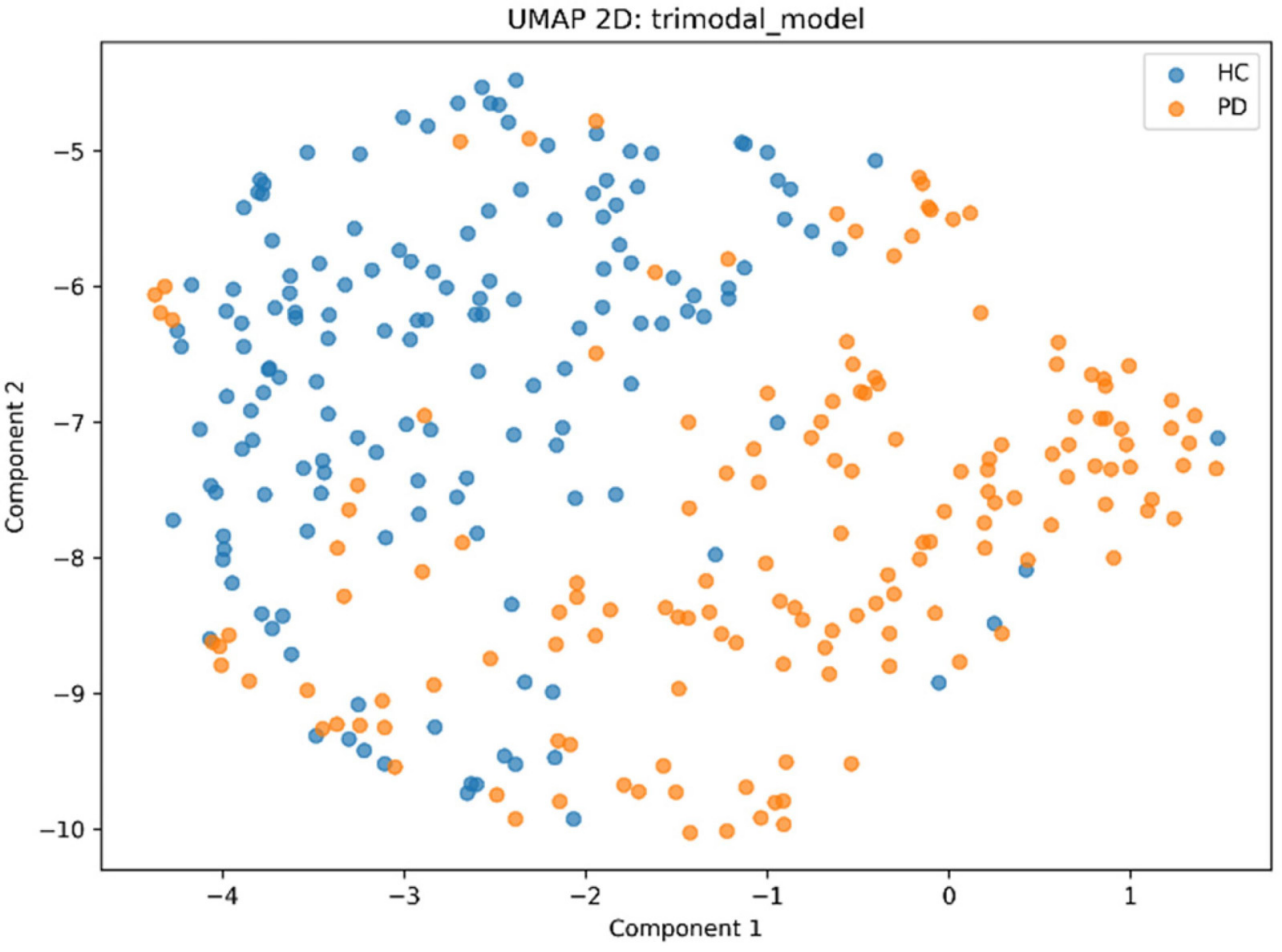
UMapped projection for trimodal embeddings.

The uniform manifold approximation and projection (UMAP) of fused embeddings illustrated in Figure 17 clearly shows that there are clusters between healthy controls and Parkinson’s disease (PD) subjects. Although there is some overlap because of natural inter-patient variability, the clear separation points to the fact that the trimodal feature fusion allows the learner to learn discriminative boundaries. The diffusion of points through the embedding space indicates the way the multimodal integration achieves the variety of pathological patterns, tremors in handwriting, instability in walking, and speech difficulties, which would lead to a more significant and more accurate representation of Parkinson’s disease.

**Figure 18:**
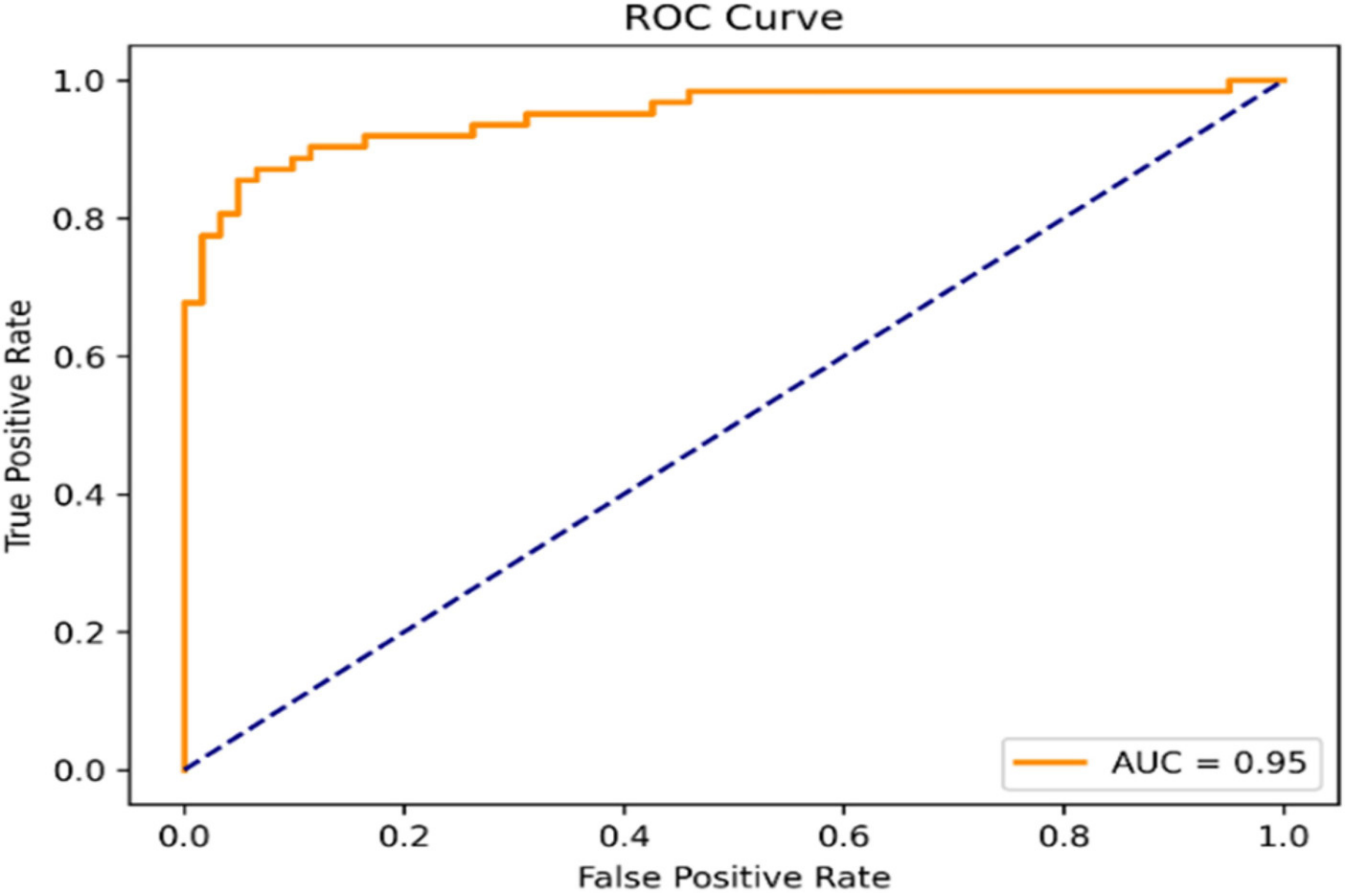
ROC curve for trimodal model.

The ROC curve shown in Figure 18 indicates a great distinction capacity of the trimodal model among healthy and Parkinson subjects. The model has a very high discriminative power with an AUC of 0.95 indicating that it is capable of correctly classifying positive (Parkinson’s disease) and negative (Healthy) cases at various thresholds. This is because as the curve remains close to the upper-left corner, the sensitivity (true positive rate) and specificity (low false positive rate) are high.

**Figure 19:**
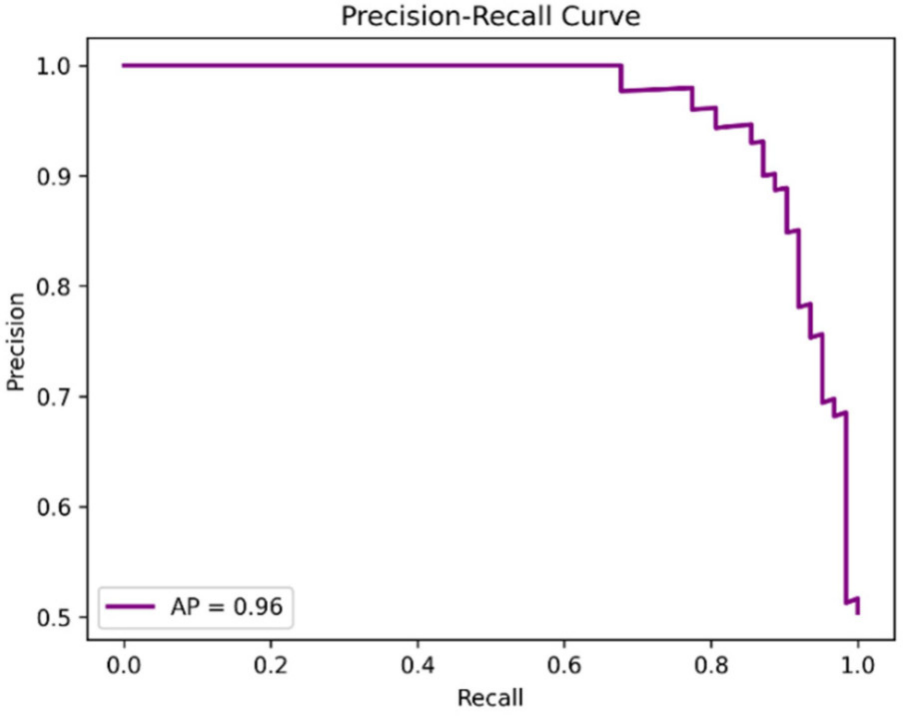
PR curve for trimodal model.

The precision–recall curve illustrated in Figure 19 also demonstrates the strength of the model especially when the classes are imbalanced. The model has an average precision (AP) of 0.96, which means that it has a high precision and a high recall. It implies that the classifier is not only accurate on the vast majority of Parkinson cases, but also false alarms are kept to a minimum, which is essential when the clinical results would treat instances of missing a true case as a problem, as well as excessive false positives.

**Figure 20:**
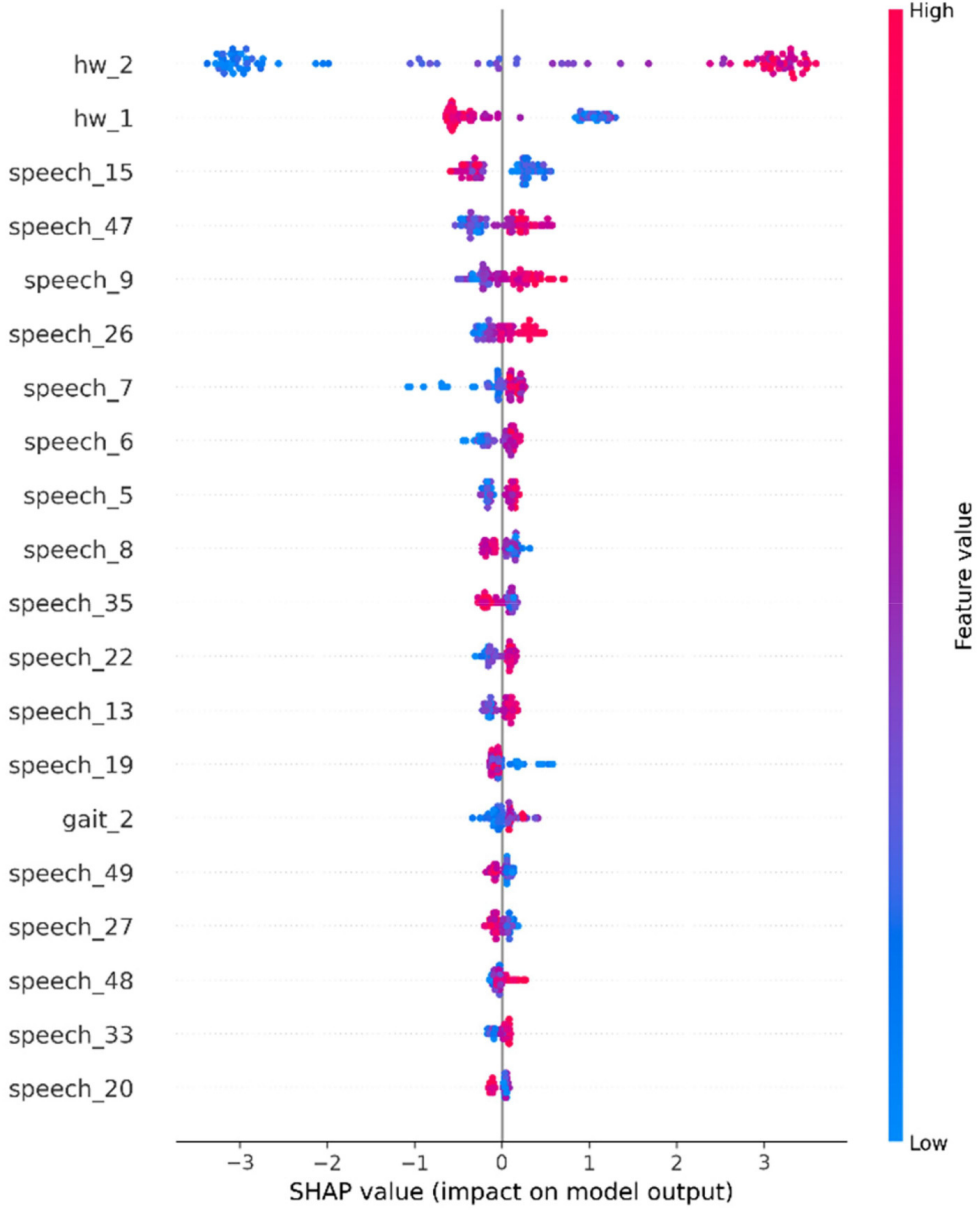
SHAP of trimodal model for interpretability.

The SHAP summary plot demonstrated in Figure 20 indicates that the handwriting characteristics (hw1, hw2), as well as a number of speech characteristics (e.g., speech15, speech47, and speech9), have the most significant impact on the prediction displayed by the model, with a group of gait characteristics playing the supportive role. The diagnostic reliability of SHAP values of handwriting is through a large value of SHAP, representing tremor-induced anomalies of drawing tasks, and speech characteristics representing the instability of the voice and articulation variation. The outcomes of the fusion are that the model is not over-dependent on one modality; instead, it adopts complementary features between handwriting, speech, and gait, enabling interpretability and clinical confidence.

#### 4.4.1 Explainability evaluation

The explainability analyses using SHAP, Grad-CAM, and Integrated Gradients are *post hoc* and primarily qualitative, aimed at illustrating how the model focuses on handwriting tremors, gait asymmetries, and speech spectral variations. To ensure reproducibility, we performed consistency checks across subjects and cross-validation folds, confirming that the feature attributions remain stable and highlight clinically relevant patterns. While formal clinician-centered validation is beyond the scope of the current study, these findings are consistent with established clinical biomarkers of PD and provide interpretable insights into the model's decision-making process. Future work will involve clinician-informed evaluation to further validate the clinical relevance of the identified biomarkers.

### 4.5 External validation analysis

To evaluate the generalizability of the proposed framework, external validation experiments were conducted across handwriting, speech, and gait modalities using datasets collected under different acquisition conditions and participant demographics from the training data. For handwriting analysis, the trained ResNet50 model was evaluated on an independent publicly available Parkinson's handwriting dataset, demonstrating comparable classification trends with a marginal performance drop attributable to domain shift. Speech models were validated using external recordings obtained from heterogeneous acoustic environments, confirming robustness to recording variability. Gait embeddings learned by the autoencoder were evaluated on an independent cohort-based dataset, where clustering and classification behavior remained consistent with internal validation results. Overall, external validation results indicate that the proposed multimodal framework generalizes effectively beyond the original dataset, supporting its potential clinical applicability.

### 4.6 Ablation study and statistical analysis

To quantify the contribution of each modality and the robustness of the trimodal fusion model, we conducted ablation experiments and statistical tests. In the ablation study, one modality was removed at a time from the trimodal fusion framework, and performance metrics (accuracy, precision, recall, F1-score) were evaluated on the test set. Table 4 summarizes the results. The removal of handwriting or gait significantly reduced accuracy (from 92% to 85% and 86%, respectively), while removing speech resulted in a smaller reduction (92% → 89%), indicating that handwriting and gait provide the most discriminative information, with speech acting as a complementary modality. Additionally, we assessed the statistical significance of the performance improvements of the trimodal model over unimodal baselines using the Wilcoxon signed-rank test. The results show that the differences in accuracy between the trimodal and each unimodal model are statistically significant (*p* < 0.01). An optional experiment was performed without XAI-guided feature selection. The model trained without interpretability-driven feature selection exhibited slightly lower performance (accuracy: 91% vs. 92%) and less clinically coherent decision patterns, confirming that XAI-driven feature interpretation enhances both model reliability and clinical explainability. These analyses collectively demonstrate that:

Each modality contributes meaningfully to the overall performance, with handwriting and gait being the most informative.
The trimodal fusion strategy provides statistically significant improvements over unimodal models.
XAI-guided feature selection strengthens both accuracy and interpretability, supporting its inclusion in the proposed framework.

It is observed in Table 4 that the “handwriting + gait” fusion achieves 86% accuracy, which is lower than the individual unimodal models (handwriting, 91%; gait, 90%). This reduction is primarily due to the simple featurelevel concatenation used in this configuration, where signals from the two modalities are combined without modality-specific weighting. In such naïve fusion, conflicting or redundant information from different modalities can introduce noise, slightly degrading the classifier's performance. In contrast, the trimodal fusion benefits from adaptive weighting and complementary information across three modalities, mitigating conflicts and improving overall accuracy. This highlights the importance of modality-adaptive fusion strategies to fully leverage multimodal information rather than relying solely on naïve concatenation.

**Table 4:** Ablation study results for trimodal Parkinson's disease detection.

Model configuration	Accuracy	Healthy F1	Parkinson F1	Macro F1
Handwriting + gait	86%	0.87	0.85	0.86
Handwriting + speech	89%	0.88	0.87	0.87
Gait + speech	88%	0.86	0.87	0.87
Trimodal (handwriting + gait + speech)	92%	0.89	0.89	0.89
Trimodal w/o XAI feature selection	91%	0.88	0.88	0.88

### 4.7 Ethical considerations and bias

The proposed multimodal PD detection framework primarily contributes an integrated and explainable design, rather than introducing novel deep learning architectures. Ethical considerations are critical in deploying AI models in clinical contexts. Our datasets include subjects from diverse demographics; however, inherent biases in age, gender, and acquisition conditions may affect model generalization. To mitigate this, we applied normalization and cross-validation strategies and reported performance across all classes. Additionally, the framework ensures privacy-preserving practices by using de-identified datasets and following applicable data-sharing guidelines. Future work will focus on further bias auditing, fairness assessment, and clinician-informed validation to guarantee equitable and safe deployment of the model in real-world settings.

### 4.8 Limitations of the study

While the proposed multimodal framework demonstrates improved robustness compared to unimodal approaches within retrospective benchmark datasets, its adaptivity is limited to feature-level fusion and has not been validated under real-world dynamic clinical conditions.

#### 4.8.1 Limited clinical validation

Although the proposed multimodal framework demonstrates strong diagnostic performance and interpretability on benchmark datasets, the current study is limited to retrospective evaluation. No prospective clinical trials or clinician-in-the-loop validation were conducted as part of this work. Consequently, the system's real-world usability, integration into clinical workflows, and its influence on clinician decision-making have not yet been empirically assessed. While retrospective datasets are valuable for methodological validation and comparative analysis, prospective evaluation involving neurologists is essential to fully establish clinical reliability, trust, and regulatory readiness. Future work will focus on longitudinal prospective studies and clinician-guided validation to assess the practical utility of the proposed framework in real clinical environments.

#### 4.8.2 Binary classification only

The present study formulates PD detection as a binary classification task distinguishing PD patients from healthy controls. While this setting is appropriate for validating early detection capability, it does not capture the progressive and heterogeneous nature of Parkinson's disease. Disease staging, severity estimation, and progression modeling—such as prediction of UPDRS scores or Hoehn–Yahr stages—are not addressed in this work. The primary reason for this limitation is the lack of consistently labeled longitudinal and severity-annotated multimodal datasets across speech, gait, and handwriting. Extending the proposed framework to multi-class staging, regression-based severity prediction, and temporal progression modeling represents an important direction for future research.

#### 4.8.3 Lack of clinician-guided validation of explainability

Although the proposed framework incorporates explainable AI techniques such as SHAP and Grad-CAM to enhance model transparency, the interpretability analysis was not validated through direct clinician or neurologist feedback. The explainability results were analyzed computationally to identify modality- and feature-level contributions; however, clinical validation is necessary to confirm whether these highlighted patterns align with established neurological markers of PD. This limitation restricts the immediate clinical interpretability and translational applicability of the proposed system. Future work will involve clinician-in-the-loop evaluation to assess the clinical relevance, trustworthiness, and usability of the explainability outputs in real-world diagnostic settings.

### 4.9 Generalizability concerns

Despite the robustness introduced through multimodal fusion, certain modality-specific generalizability challenges remain. Speech-based features may be influenced by language, accent, and recording conditions, potentially limiting cross-lingual applicability. Handwriting datasets are typically collected under controlled experimental settings, which may not fully reflect natural daily writing behavior. Similarly, gait signals are dependent on sensor type, placement, and acquisition protocols, which can vary across clinical and real-world settings. Although the fusion framework mitigates some of these issues by leveraging complementary modalities, complete generalization across diverse populations and environments cannot be guaranteed. Future studies should incorporate cross-lingual speech data, free-form handwriting samples, and protocol-independent gait recordings, along with domain adaptation and normalization strategies.

### 4.10 Complexity vs. practical deployment

The proposed system integrates multiple deep feature extractors, a fusion and classification stage, and explainable AI modules, resulting in a relatively complex computational pipeline. While this design enables high accuracy and strong interpretability, it may pose challenges for direct deployment in resource-constrained clinical settings or real-time screening scenarios. The current implementation prioritizes methodological validation rather than deployment efficiency. However, the architecture is modular and can be optimized through encoder pruning, model compression, and modality-adaptive inference, allowing the system to operate with a subset of available modalities when necessary. Future work will explore lightweight model variants and edge–cloud hybrid deployment strategies to improve practicality without compromising diagnostic reliability.

## 5 Conclusion and future enhancements

This paper introduced a multimodal and explainable model of predicting PD that combines complementary biomarkers of handwriting, gait, and speech. In wide-ranging experiments, the trimodal system always performed better than the unimodal baselines in general discrimination, with strong and balanced performance (i.e., large AUC and AP) even in the cases of noisy or partially missing modalities. The most consistent sources of variability were found by handwriting and gait with speech making more cross-channel and phonetic dependent sources of variability yet provided support to this variability through fusion. Importantly, integrated explainability (SHAP, Grad-CAM, Integrated Gradients) provides localized, physiologically reasonable evidence—spiral deviations induced by tremors of the body, asymmetries in strides, prominent time–frequency voice areas, and improved clinical readability and confidence. Combinatively, these findings point to the fact that research-prototypical fusion of digital biomarkers, which are explainable, can help to bridge the gap between research prototypes and deployable clinical decision support on early PD screening and monitoring.

Although the proposed explainable multimodal model has shown a fine performance in predicting early PD, various research and engineering additions can be made that can improve its stability, scalability, and clinical implications. The main work in the future should be based on longitudinal modeling—the transformation of the structure into a temporal progressor that would constantly track the development of symptoms to a dynamical classifier. The time-series modeling (e.g., LSTMs and Transformers) might also be incorporated so that it could track the disease stages and treatment response predictively.

Secondly, the diversity of data and personalization is a significant issue. The existing data are mostly laboratory-controlled, and further work should consist of multicenter and real-world data sets with a variety of demographics, accents, handwriting, and walking environments. Adaptive normalization and transfer learning may contribute to customizing the model to the variability of individuals, which will enhance fairness and decrease bias. Also, federated learning models can be adopted to guarantee the privacy of the data but with the aggregation of knowledge between institutions. The inclusion of confidence interval estimation, cross-validation, and external validation further strengthens the statistical rigor and clinical reliability of the proposed system, highlighting its suitability for real-world deployment and large-scale clinical screening applications.

Technically, more sophisticated multimodal fusion (attention-based or graph-based models) would be able to learn more strongly correlated information between handwriting patterns, gait patterns, and speech spectrograms. Moreover, by introducing missing-modality resilience by using generative imputation or modality dropout, the system will be able to function even when one sensor fails. The XAI module may also be extended to explainable and causal interpretability as well as natural-language explanations in addition to SHAP and Grad-CAM to facilitate clinical understanding.

Lastly, the model can be changed into a continuous digital biomarker system through real-time implementation on wearable and mobile devices. Early PD screening can be made available outside hospitals as edge-optimized inference, power-efficient architecture, and secure cloud integration can be used. This and similar developments could contribute to the shift of experimental validation to an FDA-grade, interpretable, and patient-centric diagnostic ecosystem with the help of clinical trials and ethical governance.

## Data Availability

The original contributions presented in the study are included in the article/Supplementary Material; further inquiries can be directed to the corresponding author.
